# Total Synthesis and Pharmacological Evaluation of Phochrodines A–C

**DOI:** 10.1021/acs.jnatprod.5c00104

**Published:** 2025-04-01

**Authors:** Jacob L. Bouchard, Sichen Chang, Srinivasan Krishnan, Christopher C. Presley, Olivier Boutaud, Nathan D. Schley, Darren W. Engers, Julie L. Engers, Craig W. Lindsley, Aaron M. Bender

**Affiliations:** † Warren Center for Neuroscience Drug Discovery, Vanderbilt University, Nashville, Tennessee 37232, United States; ‡ Department of Pharmacology, Vanderbilt University, Nashville, Tennessee 37232, United States; § Department of Chemistry, Vanderbilt University, Nashville, Tennessee 37240, United States; ∥ Department of Biochemistry, Vanderbilt University, Nashville, Tennessee 37205, United States

## Abstract

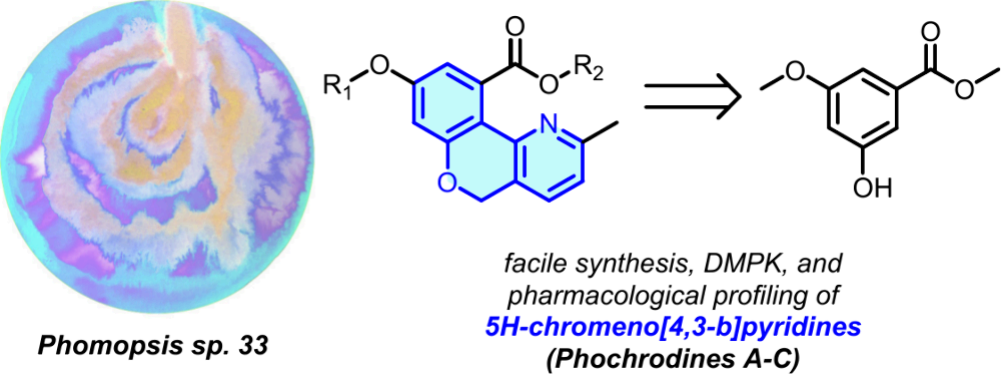

The first syntheses of the *Phomopsis*-isolated natural products phochrodines A–C are reported. Functional group manipulations on a key 5*H*-chromeno[4,3-*b*]pyridine intermediate, itself synthesized from intramolecular Suzuki–Miyaura coupling, enabled facile and high-yielding syntheses of all three natural products. Additionally, sufficient material was generated to enable detailed pharmacological profiling of each compound. Preliminary drug metabolism and pharmacokinetic (DMPK) experiments and ancillary pharmacology screening revealed phochrodine C (**3**) as an attractive scaffold for further modification, particularly for medicinal chemists working in the antidepressant space.

The identification of drug-like compounds with physicochemical and pharmacokinetic properties suitable for clinical advancement remains a significant barrier for medicinal chemists.^1^ In many drug discovery programs, a chemical series can be quickly optimized for on-target potency only to be later deprioritized due to a suboptimal DMPK or safety profile. Although there is no universal template by which to quantify the “drug-likeness” of a given compound or chemotype, a number of helpful guidelines have been widely cited and utilized by medicinal chemists.^2^

Chemotypes with suboptimal properties (metabolic liabilities, low solubility, off-target pharmacology, etc.) are often a necessary starting point for drug discovery programs, particularly for new targets or targets for which limited chemical matter has been described. Additionally, an established pharmacophore that is necessary for biological activity may prove insurmountably detrimental with respect to series development beyond simple on-target structure–activity relationship (SAR). Drug development, then, is a balancing act between on-target potency and compound properties, a scale that often tips disproportionately away from the latter.

A growing number of reports describe a more “target-agnostic” or “druggability first” approach to drug discovery. In such cases, chemotypes are developed with an eye toward efficacy or chemical property optimization prior to any knowledge of on-target SAR (as in phenotypic approaches), and there is some debate as to when in the drug discovery pipeline a target should be identified and validated.^3^ Phenotypic or target-agnostic approaches can provide the advantage of “enriched” chemical matter with which to later probe on-target SAR, although there is certainly no guarantee that this strategy will lead to the successful identification of a candidate molecule (or that the optimal properties will not erode as on-target potency increases).

Many drug-like chemical scaffolds exist in the realm of natural products, and we were interested in the identification and synthesis of a series that could serve as high-quality chemical matter for chemical optimization (outside of any knowledge of biological target). To this end, we became interested in the phochrodine natural products, an interesting series of 5*H*-chromeno[4,3-*b*]pyridines recently isolated from the mangrove endophytic fungus *Phomopsis* (Figure 1).^4^ Although it is impossible to predict the chemical properties of a chemotype with certainty *a priori*, the phochrodines represent a series of natural products with an unusually high degree of drug-likeness. For instance, with respect to Lipinski’s rule of 5 (Ro5),^2a^ Veber’s rules,^2b^ Egan’s rules,^2c^ and the Ghose filter,^2d^ all compounds within the known phochrodine series are compliant.

**Figure 1:**
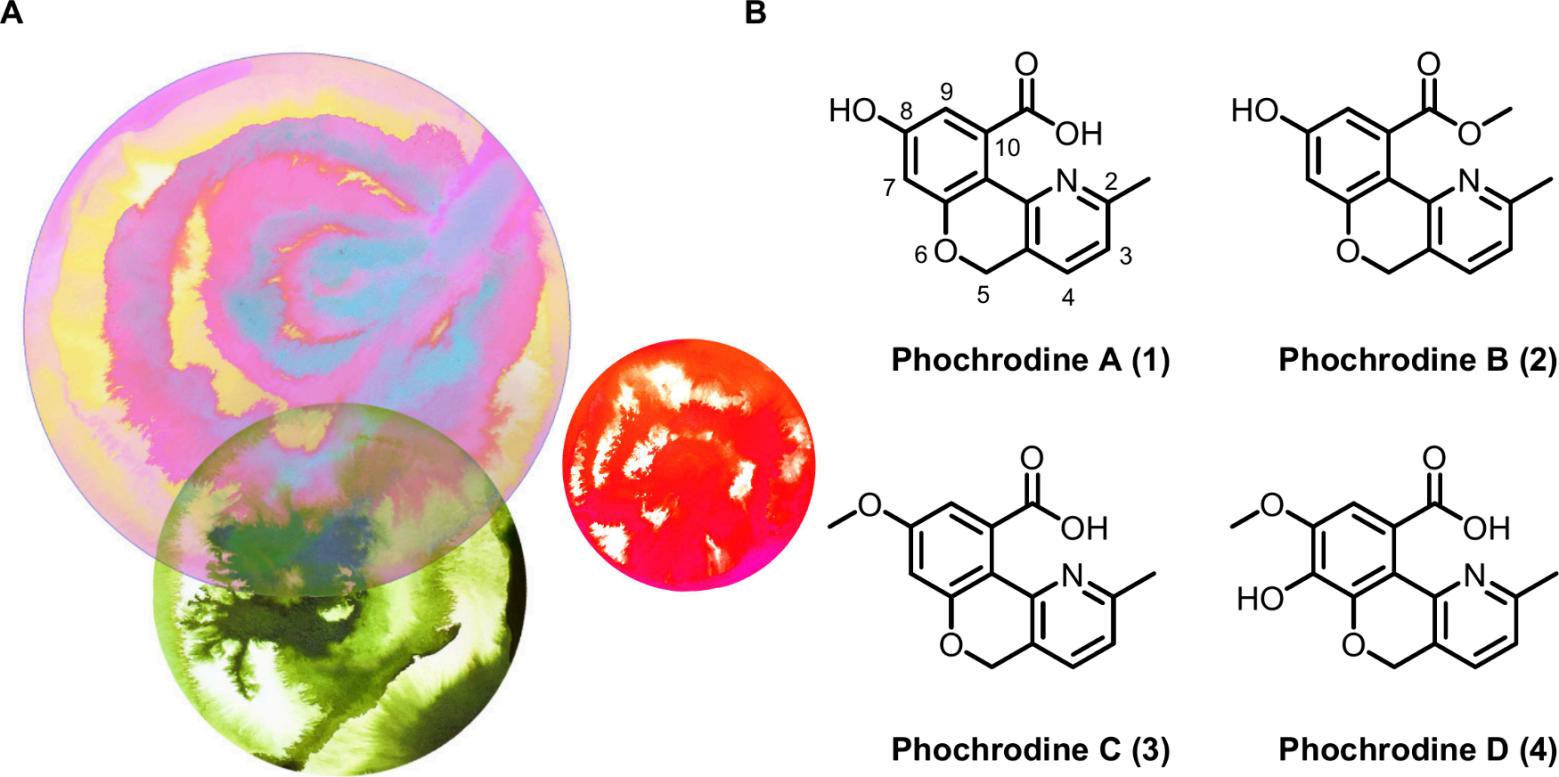
(A) Representation of *Phomopsis* and (B) chemical structures of phochrodines A–D (**1**–**4**). Artwork is courtesy of Paige Poppe.

From a synthetic point of view, the phochrodines represent the first (and to our knowledge only) known natural products featuring a 5*H*-chromeno[4,3-*b*]pyridine core,^4^ and no member of this class has yet been synthesized. We therefore endeavored to design a unified approach that would provide access to phochrodines A–C (**1**–**3**, Figure 1). Given that there are a limited number of available reports that describe the synthesis of 5*H*-chromeno[4,3-*b*]pyridines and similar tricyclic heteroarenes,^5^ we sought to develop a synthetic route that would provide not only a common intermediate toward this scaffold but one that could also be appropriately substituted at the 2, 8, and 10 positions.

## Results and Discussion

**Figure 2:**
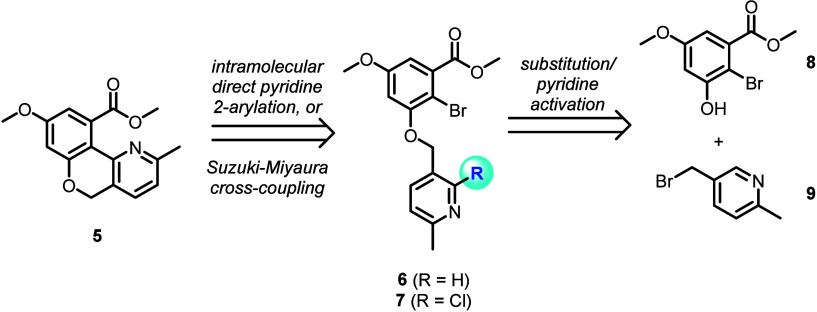
Retrosynthetic strategy to access common 5*H*-chromeno[4,3-*b*]pyridine intermediate **5**.

The proposed retrosynthesis for **1**–**3** is described in Figure 2. In short, we envisaged that key 5*H*-chromeno[4,3-*b*]pyridine intermediate **5** could provide access to phochrodines A–C through simple functional group manipulations on the methyl ester and methyl ether. Compound **5** would theoretically be accessible via cross-coupling chemistry from chloropyridine intermediate **7** (or via direct pyridine 2-arylation from intermediate **6**). The aryl ether bond of **6** could be installed through simple substitution chemistry between phenol **8** and benzyl bromide **9**.

**Scheme 1:**
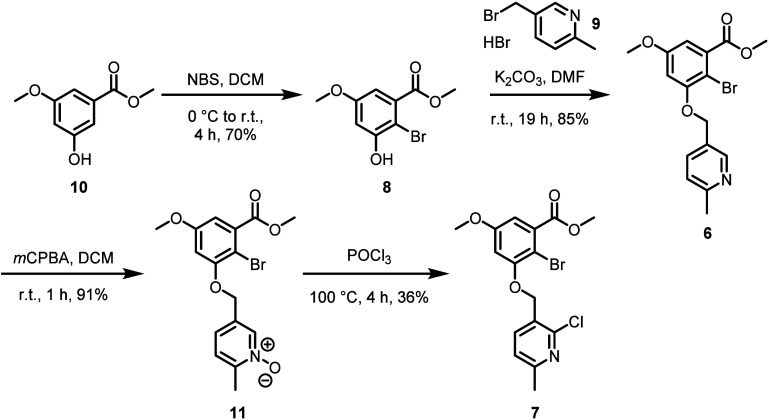
Synthesis of Suzuki–Miyaura Precursor **7**

The synthesis of arylation/cross-coupling precursor intermediates **6** and **7** is described in Scheme 1. Bromination of phenol **10** was smoothly facilitated with NBS, furnishing aryl bromide **8** in a regioselective fashion as previously described.^6a^ The observed *ortho* selectivity relative to the phenol is likely facilitated by the concerted formation of a cyclohexadienone intermediate.^6b^ Aryl ether formation with benzyl bromide **9** (as the hydrobromide salt) was also successful, providing pyridine **6** in a high yield.

At this stage, we subjected **6** to a variety of direct pyridine 2-arylation strategies that would theoretically provide 5*H*-chromeno[4,3-*b*]pyridine intermediate **5** without the need for additional pyridine functionalization steps. Unfortunately, although several such strategies are reported in the literature,^5d,7^ including Rh(I) catalysis,^7a^ direct pyridine C–H activation using a dimethyl sulfate-based transient activator approach,^7b^ and Pd-catalyzed arylations of azine/azole *N*-oxides,^7c^ all attempted conditions failed to give **5** in this context. In general, reports of direct pyridine 2-arylations are typically limited to *intermolecular* transformations on simple pyridines and often require large excesses of one coupling partner.^7^ Additionally, the 2-pyridine position of **6** may not be predisposed to approach the metal-inserted C–Br bond. With the knowledge that pyridine prefunctionalization would therefore likely be necessary to achieve the desired cyclization, we first prepared pyridine *N*-oxide **11**, followed by chlorination to give key chloropyridine intermediate **7** (Scheme 1). Although the yield for the chlorination step was modest (presumably due to competing pyridine 4-chlorination), **7** was readily isolated via column chromatography.

**Scheme 2:**
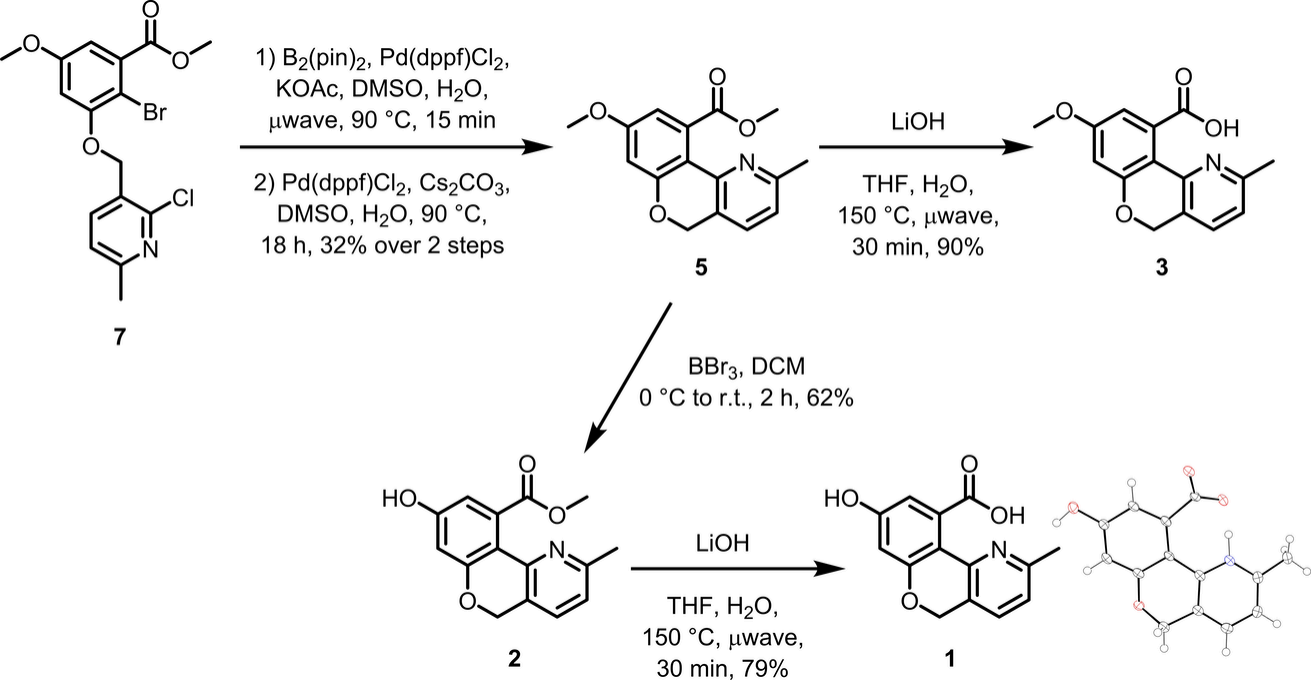
Synthesis of Phochrodines A–C (**1–3**)

We were ultimately gratified to find that **7** could be converted to cyclic intermediate **5** over a 2-step sequence: (1) conversion of the aryl bromide to a pinacol boronic ester species, and (2) subjection to classical Suzuki–Miyaura conditions to give **5** in moderate yield (Scheme 2). Although complete conversion of pinacol boronate was observed in the Suzuki–Miyaura reaction, the modest overall yield is likely due to the competing dehalogenation observed in the borylation step. From **5**, BBr_3_-mediated demethylation gave phochrodine B (**2**); subsequent methyl ester hydrolysis afforded phochrodine A (**1**). Direct methyl ester hydrolysis of intermediate **5** gave phochrodine C (**3**). (In the case of **1** and **3**, strongly forcing hydrolysis conditions proved necessary for complete reactivity; see Experimental Section for further synthetic details.) This synthetic route ultimately furnished sufficient material of **1**–**3** for further evaluation.

The spectral data for **2** and **3** matched those of the natural isolates in all respects. In the case of phochrodine A (**1**), although the chemical shifts in both the ^1^H and ^13^C NMR spectra were in good agreement, small discrepancies were noted for the aromatic *J*-couplings in the ^1^H NMR spectra (7.8 Hz (*ortho*) and 2.7 Hz (*meta*) for the synthetic material, vs 5.2 and 1.6 Hz reported for the natural isolate).^4^ The structure of **1** was subsequently confirmed by X-ray analysis and was found to be identical with the reported structure for phochrodine A (see the Supporting Information for further details).

With **1**–**3** in hand, we next turned our attention to profiling the pharmacokinetics of each natural product. Gratifyingly, in our standard *in vitro* predicted clearance assays, natural products **1** and **3** displayed low turnover in both human and rat microsomes (Table 1). By contrast, compound **2** showed higher turnover in both species, presumably due to esterase-mediated cleavage of the methyl ester motif. Phochrodines A–C (**1**–**3**) displayed excellent free fraction in human plasma samples (>10% unbound drug), with lower free drug levels observed in rat (>4% unbound).^8^ In human P-glycoprotein (P-gp)-transfected MDCKII-MDR1 cells, **1**–**3** all displayed efflux ratios <2, indicating a high probability of CNS penetration in humans. Compounds **2** and **3** were also found to be highly membrane permeable (*P*_app_,_A–B_ >90 × 10^–6^ cm/s; see Table 2). Additionally, **1**–**3** all displayed excellent solubility in our kinetic solubility assay at both acidic and nearly neutral pH (>78 μM for all compounds, see Table 1),^9^ and compound **2** was within the typical range for drug-like compounds in the octanol–water distribution assay (ELog*D*_7.4_ of 2.4, see Table 1).^10^ Encouraged by the full package of *in vitro* properties observed for carboxylic acid analogs **1** and **3**, particularly the high predicted permeability for compound **3**, we selected phochrodine C (**3**) for pharmacological profiling.

**Table 1:** Drug Metabolism and Pharmacokinetic Properties of **1–3**

Phochrodine	CL_hep_ ((mL/min)**/**kg)^a^	PPB^b^	Kinetic Solubility (μM)^c^	ELog*D*_7**.**4_ (XLog*P*)^d^
A (**1**)	5.3 (h),	0.46 (h),	98.3 (pH = 2.2),	-
	4.9 (r)	0.14 (r)	>100 (pH = 6.8)	(1.2)
B (**2**)	16.2 (h),	0.13 (h),	96.2 (pH = 2.2),	2.4
	54.9 (r)	0.04 (r)	78.2 (pH = 6.8)	(1.9)
C (**3**)	5.8 (h),	0.35 (h),	89.9 (pH = 2.2),	-
	9.7 (r)	0.07 (r)	94.9 (pH = 6.8)	(1.6)

a Predicted hepatic clearance (microsomes).

b Plasma protein binding (*f*_u_) via equilibrium dialysis.

c Aqueous kinetic solubility.

d Chromatographic method is not suitable for the extrapolation of acidic compounds. See the Experimental Section for further details regarding all DMPK data. h = human, r = rat.

**Table 2:** Apparent Permeability and Efflux Ratios for Compounds Tested in Human P-gp-Transfected MDCKII-MDR1 Cells^a^

Phochrodine	*P*_app(A-to-B)_ (10^**–**6^ cm/s)	Efflux Ratio
A (**1**)	9.13 ± 0.39	1.11 ± 0.01
B (**2**)	104 ± 7.75	1.27 ± 0.06
C (**3**)	91.5 ± 4.75	1.65 ± 0.09

a Results are *n* = 2, ±SEM. See the Experimental Section for further details.

In an ancillary pharmacology screen of 44 targets (Eurofins Cerep),^11^ compound **3** was found to have <50% inhibition for all targets at 10 μM, with the exception of monoamine oxidase A (MAO-A) (57%, see Table 3). These results are noteworthy for two reasons: (1) the finding that phochrodine C (**3**) is clean with respect to key drug discovery antitargets including the hERG channel, the serotonin receptor subtype 2B (5-HT_2B_), the mu opioid receptor (MOR), and the dopamine transporter (DAT),^12^ and (2) the finding that **3** is (weakly) active at MAO-A, indicating that this scaffold could serve as a starting point for medicinal chemists looking to develop differentiating or next-generation monoamine oxidase inhibitors (MAOIs). Although MAOIs are generally not the first-in-class treatment for depression and related mood disorders (their use has been largely supplanted by the selective serotonin reuptake inhibitors (SSRIs) due to safety concerns with the former), several MAOIs, particularly the reversible inhibitors, still find some clinical use today.^13^ Moreover, the success of medicinal chemistry programs demands the identification of novel heteroaryl systems to differentiate from well-trodden and overused chemotypes.^14^ The favorable PK and clean ancillary pharmacology data presented herein suggest that the tricyclic 5*H*-chromeno[4,3-*b*]pyridine scaffold could be broadly integrated in drug discovery programs.

**Table 3:** Percent Inhibition of Radioligand Binding for Compound **3** at 10 μM^a^

Target	% Inhibition (10 μM)
sodium channel site 2 (nonselective)	–2
A_2A_ (h)	4
alpha_1A_ (h)	–2
alpha_2A_ (h)	6
beta_1_ (h)	–2
beta_2_ (h)	8
BZD (central)	11
CB_2_ (h)	9
CB_1_ (h)	6
CCK_1_ (CCK_A_) (h)	10
D_1_ (h)	–4
D_2S_ (h)	–9
ET_A_ (h)	8
NMDA	6
H_1_ (h)	–1
H_2_ (h)	–6
MAO-A	**57**
M_1_ (h)	–5
M_2_ (h)	–3
M_3_ (h)	6
N neuronal α4β2 (h)	–6
DOR (h)	–1
KOR (h)	–3
MOR (h)	4
5-HT_1A_ (h)	9
5-HT_1B_ (h)	0
5-HT_2A_ (h)	7
5-HT_2B_ (h)	16
5-HT_3_ (h)	7
GR (h)	1
AR (h)	–10
V_1a_ (h)	–1
Ca^2+^ channel (L dihydropyridine site)	6
hERG (h)	14
K_V_ channel	2
norepinephrine transporter (h)	7
dopamine transporter (h)	–0.5
5-HT transporter (h)	1
LcK TK kinase (h)	–7
COX-1 (h)	18
COX-2 (h)	–10
PDE3A (h)	0.2
PDE4D2 (h)	–24
AChE (h)	–6

a (h) = human. See ref (11).

In conclusion, we have accomplished the first syntheses of the mangrove endophytic fungus-derived natural products phochrodines A–C. All compounds in this series were accessed from an intramolecular borylation/Suzuki–Miyaura cyclization that yielded the key 5*H*-chromeno[4,3-*b*]pyridine intermediate **5**, from which functional group manipulations afforded natural products **1**–**3**. As a starting point for medicinal chemistry, **1**–**3** represent an intriguing class of natural products with an unusually high degree of drug-likeness as determined by *in vitro* microsomal clearance, plasma protein binding, P-gp efflux, and solubility. Ancillary pharmacology screening of compound **3** revealed a clean profile on key antitargets with weak activity observed for MAO-A. A large share of natural products (biogenic-type amines, polyphenols, etc.) are promiscuous with respect to biological targets. By contrast, the clean ancillary pharmacology of phochrodine C (**3**) is a relative rarity among natural products and thus provides an exciting template for additional SAR (MAOI or otherwise). Synthetic compounds featuring a substituted 5*H*-chromeno[4,3-*b*]pyridine scaffold have also been previously reported as kinase^15^ and topoisomerase^16^ inhibitors, and it is our hope that follow-up studies on the phochrodines and their synthetic derivatives will reveal additional pharmacology and opportunities for medicinal chemistry.

## Experimental Section

### General Experimental Procedures

All reactions were carried out by employing standard chemical techniques. Solvents used for the reactions and extraction were ACS grade, and HPLC grade solvents were used for purification. All reagents were purchased from commercial sources and were used without further purification.

All NMR spectra were recorded on a 400 MHz Bruker AV-400 instrument. ^1^H chemical shifts are reported as δ values in ppm relative to the residual solvent peak (CDCl_3_ = 7.26, CD_3_OD = 3.31). Data are reported as follows: chemical shift, multiplicity (br = broad, s = singlet, d = doublet, t = triplet, q = quartet, p = pentet, dd = doublet of doublets, ddd = doublet of doublet of doublets, td = triplet of doublets, m = multiplet), coupling constant, and integration. ^13^C chemical shifts are reported as δ values in ppm relative to the residual solvent peak (CDCl_3_ = 77.16, CD_3_OD = 49.00).

LCMS data were obtained on a Waters QDa (Performance) SQ MS instrument with ESI source. MS parameters were as follows: cone voltage: 15 V, capillary voltage: 0.8 kV, probe temperature: 600 °C. Samples were introduced via an Acquity I-Class PLUS UPLC comprised of a BSM, FLSM, CH-A, and PDA. UV absorption was generally observed at 215 and 254 nm; 4 nm bandwidth. Column: Phenomenex EVO C18, 1.0 mm × 50 mm, 1.7 μm. Column temperature: 55 °C. Flow rate: 0.4 mL/min. Default gradient: 5% to 95% CH_3_CN (0.05% TFA) in water (0.05% TFA) over 1.4 min, hold at 95% CH_3_CN for 0.1 min.

High-resolution mass spectra were obtained on an Agilent 6540 UHD Q-TOF with an ESI source. MS parameters were as follows: fragmentor: 150, capillary voltage: 3500 V, nebulizer pressure: 60 psig, drying gas flow: 13 L/min, drying gas temperature: 275 °C. Samples were introduced via an Agilent 1290 UHPLC comprising a G4220A binary pump, G4226A ALS, G1316C TCC, and G4212A DAD with ULD flow cell. UV absorption was observed at 215 and 254 nm with a 4 nm bandwidth. Column: Agilent Zorbax Extend C18, 1.8 μm, 2.1 mm × 50 mm. Gradient conditions: 5% to 95% CH_3_CN in water (0.1% formic acid) over 1 min, hold at 95% CH_3_CN for 0.1 min, 0.5 mL/min, 40 °C.

Automated flash column chromatography was performed on a Biotage Isolera 1 or Teledyne ISCO CombiFlash system. Microwave synthesis was performed in a Biotage Initiator microwave synthesis reactor. Melting points were recorded on an OptiMelt automated melting point system from Stanford Research Systems.

### Safety Statement

*No unexpected or unusually high safety hazards were encountered*.

### Chemistry

#### Methyl 2-Bromo-3-hydroxy-5-methoxybenzoate (**8**)

A solution of methyl 3-hydroxy-5-methoxybenzoate (**10**) (300 mg, 1.65 mmol, 1 equiv) in DCM (6 mL) was cooled to 0 °C and stirred for 5 min. *N*-Bromosuccinimide (308 mg, 1.73 mmol, 1.05 equiv) was then added in one portion. The resulting solution was warmed to rt and stirred for 4 h, after which time a saturated NaHCO_3_ solution was added. The aqueous layer was extracted with DCM, and combined organic extracts were dried over MgSO_4_, filtered, and concentrated. The crude residue was purified by column chromatography (3–20% EtOAc in hexanes) to give the title compound as a colorless oil (201 mg, 70%): ^1^H NMR (400 MHz, CDCl_3_) δ 7.00 (d, *J* = 3.0 Hz, 1H), 6.75 (d, *J* = 3.0 Hz, 1H), 6.01 (s, 1H), 3.92 (s, 3H), 3.81 (s, 3H); ^13^C{^1^H} NMR (101 MHz, CDCl_3_) δ 166.3, 159.8, 154.1, 132.4, 110.0, 104.9, 100.9, 55.9, 52.7; HRMS (TOF, ESI) calcd for C_9_H_10_BrO_4_ [M + H]^+^ = 260.9757, found = 260.9752. Analytical and spectral data match those previously reported.^6^

#### Methyl 2-Bromo-5-methoxy-3-((6-methylpyridin-3-yl)methoxy)benzoate (**6**)

Compound **8** (626 mg, 2.40 mmol, 1 equiv), 5-(bromomethyl)-2-methylpyridine hydrobromide (**9**) (704 mg, 2.64 mmol, 1.1 equiv), and potassium carbonate (841 mg, 6.00 mmol, 2.5 equiv) were combined in DMF (12 mL), and the resulting reaction mixture was stirred at rt under an inert atmosphere for 19 h, after which time the reaction mixture was diluted with brine and EtOAc. The organic layer was washed with brine, dried over MgSO_4_, filtered, and concentrated. The crude residue was purified by column chromatography (3–70% EtOAc in hexanes) to give the title compound as a white solid (746 mg, 85%): ^1^H NMR (400 MHz, CDCl_3_) δ 8.58 (d, *J* = 1.7 Hz, 1H), 7.73 (dd, *J* = 8.0, 2.3 Hz, 1H), 7.20 (d, *J* = 8.0 Hz, 1H), 6.84 (d, *J* = 2.8 Hz, 1H), 6.63 (d, *J* = 2.8 Hz, 1H), 5.10 (s, 2H), 3.93 (s, 3H), 3.80 (s, 3H), 2.57 (s, 3H); ^13^C{^1^H} NMR (101 MHz, CDCl_3_) δ: 167.2, 159.6, 158.7, 156.1, 148.2, 135.7, 135.1, 128.6, 123.4, 107.2, 104.3, 103.1, 69.1, 55.9, 52.8, 24.4; HRMS (TOF, ESI) calcd for C_16_H_17_BrNO_4_ [M + H]^+^ = 366.0335, found = 366.0332. Melting point: 105–107 °C.

#### 5-((2-Bromo-5-methoxy-3-(methoxycarbonyl)phenoxy)methyl)-2-methylpyridine 1-Oxide (**11**)

To a solution of **6** (397 mg, 1.08 mmol, 1 equiv) in DCM (5 mL) was added *m*CBPA (728 mg, 3.25 mmol, 3 equiv) in one portion. The resulting reaction mixture was stirred at rt for 1 h, after which time the reaction mixture was diluted with a saturated NaHCO_3_ solution and extracted with DCM. Combined organic extracts were dried over MgSO_4_, filtered, and concentrated. The crude residue was purified by column chromatography (3–100% EtOAc in hexanes and then 0–7% MeOH in DCM) to give the title compound as a white solid (378 mg, 91%): ^1^H NMR (400 MHz, CDCl_3_) δ 8.43–8.40 (m, 1H), 7.32–7.29 (m, 2H), 6.86 (d, *J* = 2.8 Hz, 1H), 6.58 (d, *J* = 2.7 Hz, 1H), 5.05 (s, 2H), 3.94 (s, 3H), 3.81 (s, 3H), 2.53 (s, 3H); ^13^C{^1^H} NMR (101 MHz, CDCl_3_) δ 167.1, 159.6, 155.6, 148.7, 138.0, 135.3, 133.1, 126.6, 124.2, 107.5, 104.3, 103.1, 67.7, 55.9, 52.8, 17.8; HRMS (TOF, ESI) calcd for C_16_H_17_BrNO_5_ [M + H]^+^ = 382.0285, found = 382.0280. Melting point: 121–123 °C.

#### Methyl 2-Bromo-3-((2-chloro-6-methylpyridin-3-yl)methoxy)-5-methoxybenzoate (**7**)

Compound **11** (367 mg, 0.96 mmol, 1 equiv) and phosphorus(V) oxychloride (4.49 mL, 48.0 mmol, 50 equiv) were combined and heated to 100 °C for 4 h, after which time the reaction mixture was cooled to rt and poured into a cold saturated NaHCO_3_ solution. The aqueous layer was extracted with EtOAc, and combined organic extracts were dried over MgSO_4_. Solvents were filtered and concentrated, and the crude residue was purified by column chromatography (3–60% EtOAc in hexanes) to give the title compound as a white solid (137 mg, 36%): ^1^H NMR (400 MHz, CDCl_3_) δ 7.97 (dd, *J* = 7.7, 0.9 Hz, 1H), 7.17 (d, *J* = 7.7 Hz, 1H), 6.86 (d, *J* = 2.8 Hz, 1H), 6.65 (d, *J* = 2.8 Hz, 1H), 5.15 (s, 2H), 3.95 (s, 3H), 3.82 (s, 3H), 2.55 (s, 3H); ^13^C{^1^H} NMR (101 MHz, CDCl_3_) δ 167.2, 159.7, 158.8, 155.8, 147.7, 137.6, 135.1, 127.5, 122.7, 107.3, 103.9, 102.8, 67.2, 56.0, 52.8, 24.0; HRMS (TOF, ESI) calcd for C_16_H_16_BrClNO_4_ [M + H]^+^ = 399.9946, found = 399.9940. Melting point: 120–123 °C.

#### Methyl 8-Methoxy-2-methyl-5*H*-chromeno[4,3-*b*]pyridine-10-carboxylate (**5**)

To a vial containing compound **7** (25 mg, 0.062 mmol, 1 equiv) in DMSO (0.5 mL) was added water (0.05 mL), bis(pinacolato)diboron (31.7 mg, 0.12 mmol, 2 equiv), potassium acetate (18 mg, 0.19 mmol, 3 equiv), and Pd(dppf)Cl_2_ (14 mg, 0.019 mmol, 0.3 equiv). The resulting reaction mixture was degassed with N_2_ for 10 min, sealed, and heated to 90 °C for 15 min under microwave irradiation. The reaction mixture was cooled to rt, filtered through a pad of Celite, passed through a hydrophobic phase separator, and concentrated to afford the pinacol boronic ester intermediate, which was taken directly to the next step without further purification (assumed quantitative yield for subsequent step despite competing dehalogenation reaction). To a vial containing the pinacol boronic ester intermediate (28 mg, 0.062 mmol, 1 equiv) in DMSO (0.5 mL) were added water (0.05 mL), cesium carbonate (41 mg, 0.12 mmol, 2 equiv), and Pd(dppf)Cl_2_ (14 mg, 0.019 mmol, 0.3 equiv). The resulting reaction mixture was degassed with N_2_ for 10 min and then sealed and heated to 90 °C for 18 h. The reaction mixture was cooled to rt and filtered through a pad of Celite. DCM and H_2_O were added, and the aqueous layer was extracted with DCM. Combined organic extracts were passed through a hydrophobic phase separator and concentrated. The crude residue was purified by column chromatography (0–100% EtOAc in hexanes) to give the title compound as an oil (5.7 mg, 32% over two steps): ^1^H NMR (400 MHz, CD_3_OD) δ 7.47 (d, *J* = 7.8 Hz, 1H), 7.08 (d, *J* = 7.7 Hz, 1H), 6.70 (d, *J* = 2.5 Hz, 1H), 6.65 (d, *J* = 2.5 Hz, 1H), 5.15 (s, 2H), 3.84 (s, 3H), 3.84 (s, 3H), 2.48 (s, 3H); ^13^C{^1^H} NMR (101 MHz, CD_3_OD) δ: 172.7, 163.1, 160.0, 158.7, 148.0, 134.2, 133.7, 123.9, 122.4, 115.5, 110.0, 104.3, 69.2, 56.2, 52.9, 24.4; HRMS (TOF, ESI) calcd for C_16_H_16_NO_4_ [M + H]^+^ = 286.1074, found = 286.1075.

#### 8-Hydroxy-2-methyl-5*H*-chromeno[4,3-*b*]pyridine-10-carboxylic Acid (**1**)

To a solution of compound **2** (300 mg, 1.11 mmol, 1 equiv) in THF (4 mL) was added an aqueous 1 M lithium hydroxide solution (6.64 mL, 6.64 mmol, 6 equiv). The resulting reaction mixture was heated under microwave irradiation for 30 min at 150 °C. Upon completion, the pH of the solution was adjusted to 5 with a 2 M aqueous HCl solution and extracted with EtOAc. The combined organic extracts were passed through a hydrophobic phase separator and concentrated. The crude residue was purified by column chromatography (0–100% EtOAc in hexanes) to give the title compound as a white solid (224 mg, 79%): ^1^H NMR (400 MHz, CD_3_OD) δ 7.86 (d, *J* = 7.8 Hz, 1H), 7.37 (d, *J* = 7.8 Hz, 1H), 7.24 (d, *J* = 2.7 Hz, 1H), 6.58 (d, *J* = 2.6 Hz, 1H), 5.14 (s, 2H), 2.64 (s, 3H); ^13^C{^1^H} NMR (101 MHz, CD_3_OD) δ 173.4, 162.7, 161.2, 154.5, 146.8, 138.2, 138.1, 126.7, 123.9, 116.5, 110.5, 107.4, 67.7, 21.1; HRMS (TOF, ESI) calcd for C_14_H_12_NO_4_ [M + H]^+^ = 258.0761, found 258.0763. Melting point not determined; decomp < 300 °C.

#### Methyl 8-Hydroxy-2-methyl-5*H*-chromeno[4,3-*b*]pyridine-10-carboxylate (**2**)

To a solution of compound **5** (14 mg, 0.05 mmol, 1 equiv) in DCM (0.5 mL) was added a boron tribromide solution (0.098 mL, 0.098 mmol, 2 equiv, 1 M solution in DCM) via syringe dropwise at 0 °C. Upon completion of addition, the reaction mixture was stirred at rt for 2 h. The reaction mixture was then diluted with DCM and quenched with water. The resulting layers were separated, and the organic layer was passed through a hydrophobic phase separator. The organic layer was then concentrated and purified by column chromatography (0–100% EtOAc in hexanes) to give the title compound as a yellow-orange solid (8.2 mg, 62%): ^1^H NMR (400 MHz, CDCl_3_) δ 7.32 (d, *J* = 7.7 Hz, 1H), 6.98 (d, *J* = 7.7 Hz, 1H), 6.68 (d, *J* = 2.4 Hz, 1H), 6.53 (d, *J* = 2.4 Hz, 1H), 5.85 (br s, 1H), 5.12 (s, 2H), 3.89 (s, 3H), 2.52 (s, 3H); ^13^C{^1^H} NMR (101 MHz, CDCl_3_) δ 170.9, 158.6, 158.2, 157.5, 146.7, 132.9, 132.7, 122.3, 121.4, 110.4, 105.6, 68.2, 52.7, 24.5. *Note*: one quaternary aromatic signal is obscured in the CDCl_3_^13^C NMR spectrum; an additional spectrum in CD_3_OD confirms the presence of 12 aromatic signals: ^13^C{^1^H} NMR (101 MHz, CD_3_OD) δ 174.5, 172.3, 160.4, 158.0, 150.0, 133.6, 133.1, 123.1, 120.1, 115.8, 109.1, 108.1, 68.9, 52.6, 24.5; HRMS (TOF, ESI) calcd for C_15_H_14_NO_4_ [M + H]^+^ = 272.0917, found 272.0917. Melting point: 171–175 °C. Spectral data match those previously reported.^4^

#### 8-Methoxy-2-methyl-5*H*-chromeno[4,3-*b*]pyridine-10-carboxylic Acid (**3**)

To a solution of compound **5** (25 mg, 0.09 mmol, 1 equiv) in THF (0.5 mL) was added an aqueous 1 M lithium hydroxide solution (0.5 mL, 0.5 mmol, 5.7 equiv). The resulting reaction mixture was heated under microwave irradiation for 30 min at 150 °C. Upon completion, the pH of the solution was adjusted to 5 with a 2 M aqueous HCl solution and extracted with EtOAc. The combined organic extracts were passed through a hydrophobic phase separator and concentrated. The crude residue was purified by column chromatography (0–100% EtOAc in hexanes) to give the title compound as a light yellow solid (22 mg, 90%): ^1^H NMR (400 MHz, CDCl_3_) δ 7.74 (d, *J* = 2.8 Hz, 1H), 7.65 (d, *J* = 7.7 Hz, 1H), 7.23 (d, *J* = 7.7 Hz, 1H), 6.73 (d, *J* = 2.8 Hz, 1H), 5.08 (s, 2H), 3.89 (s, 3H), 2.68 (s, 3H); ^13^C{^1^H} NMR (101 MHz, CDCl_3_) δ 169.0, 162.2, 159.3, 154.2, 146.7, 135.8, 135.6, 125.1, 122.8, 115.9, 112.3, 106.9, 66.9, 55.9, 21.9; HRMS (TOF, ESI) calculated for C_15_H_14_NO_4_ [M + H]^+^ = 272.0917, found 272.0920. Melting point: 156–158 °C. Spectral data match those previously reported.^4^

### Experimental Procedures (DMPK)

#### Plasma Protein Binding

The plasma protein biding assay for human and rat was conducted as previously described.^8^ Determination of fraction unbound (*f*_u_) in plasma from rat and human was conducted *in vitro* via equilibrium dialysis using HTDialysis membrane plates. The top half of the plate was filled with 100 μL of Dulbecco’s phosphate buffered saline, pH 7.4 (DPBS). Compound was diluted into plasma from each species (5 μM final concentration), which was aliquoted in triplicate to the “bottom half” of the prepared HTD plate wells. The HTD plate was sealed and incubated for 6 h at 37 °C. Following incubation, each well (both top and bottom halves) was transferred (20 μL) to the corresponding wells of a 96-shallow-well (V-bottom) plate. The daughter plates were then matrix-matched (DPBS side wells received an equal volume of plasma, and plasma side wells received an equal volume of DPBS), and extraction solution (120 μL; acetonitrile containing 50 nM carbamazepine as IS) was added to all wells of both daughter plates to precipitate protein and extract test article. The plates were then sealed and centrifuged (3500 rcf) for 10 min at ambient temperature. Supernatant (60 μL) from each well of the daughter plates was then transferred to the corresponding wells of new daughter plates (96-shallow-well, V bottom) containing water (Milli-Q, 60 μL/well), and the plates were sealed in preparation for LC-MS/MS analysis as follows.

Prepared samples were injected (10 μL each) into an AB Sciex Triple Quad 4500 mass spectrometer system with an Agilent 1260 Infinity II pump and autosampler. MS parameters were as follows: capillary voltage: 5500 V, probe temperature: 500 °C. Column: Fortis C18 (50 mm × 3.0 mm, 3 μm). Column temperature: 45 °C. Flow rate: 0.5 mL/min. Default gradient: 5% to 95% CH_3_CN (0.5% FA) in water (0.5% FA) over 0.8 min, hold at 95% CH_3_CN for 0.7 min. Quantitation was performed via AB Sciex Multiquant software using the raw analyte:IS peak area ratios. The typical detection range for the compounds was 0.5 ng/mL to ≥5000 ng/mL utilizing a quadratic equation regression with 1/*x*2 weighting.

The unbound fraction (*f*_u_) was calculated following the equation [mean DPBS well ratio/mean plasma well ratio], and mean values for each species were calculated from 3 replicates.

#### Predicted Microsomal Clearance

Human, rat, and mouse hepatic microsomes (0.5 mg/mL) and 1 μM test compound were incubated in 100 mM potassium phosphate (pH 7.4) buffer with 3 mM MgCl_2_ at 37 °C with constant shaking. After a 5 min preincubation, the reaction was initiated by addition of NADPH (1 mM). At selected time intervals (0, 3, 7, 15, 25, and 45 min), aliquots were taken and subsequently placed into a 96-well plate containing cold acetonitrile with internal standard (50 nM carbamazepine). Plates were then centrifuged at 3000 RCF (4 °C) for 10 min, and the supernatant was transferred to a separate 96-well plate and diluted 1:1 with water for LC/MS/MS analysis. The *in vitro* half-life (*t*_1/2_, min), intrinsic clearance (CL_int_, mL/min/kg), and subsequent predicted hepatic clearance (CL_hep_, mL/min/kg) were determined using eqs 1–3:
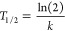
1Equation 1 is the determination of the half-life. *k* represents the slope from linear regression analysis of the natural log percent remaining of the test compound as a function of incubation time.
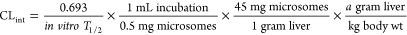
2Equation 2 is the determination of the intrinsic clearance. Scale-up factors (gm liver/kg body weight) of 20 (human) and 45 (rat) were used in this calculation (scaling factors were derived from Lin et al.^17^).
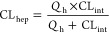
3Equation 3 is the determination of the predicted hepatic clearance. *Q*_h_ represents hepatic blood flow (mL/min/kg): 21 for human, 70 for rat.

#### Kinetic Solubility

An adapted standard shake flask method was run in 1 mL 96-deep-well plates at a concentration of 100 μM in McIlvaine buffer at pH values of 2.2 and 6.8 from 10 mM DMSO stock solutions. Compounds are prepared in triplicate and incubated in buffer at room temperature for 18 h while shaking at 700 rpm. After incubation the 96-deep-well plate is centrifuged at 5000*g* for 10 min, and half of the volume is transferred to another deep-well plate and centrifuged again at 5000*g* for 10 min. A 200 μL amount is transferred from each well to a Greiner Bio-one 200 μL 96-well V-bottom plate and sealed. A six-point calibration curve is prepared for each compound ranging from 100 μM down to 0.5 μM. All samples are analyzed via UV-UHPLC on an Agilent 1290 Infinity (binary pump, autosampler, column compartment at 55 °C, and PDA) with a Phenomenex Kinetex EVO C18, 50 × 1 mm, 1.7 μm, 100 Å column, at 0.5 mL/min. Injections of 3 μL are analyzed with gradient elution using Milli-Q water with 0.05% trifluoroacetic acid (A1) and acetonitrile with 0.05% trifluoroacetic acid (B1) from 95:5 A1/B1 to 5:95 A1/B1 over 1.3 min with a 0.2 min hold at 5:95 A1/B1. Wavelengths at 215 and 254 nm are monitored, peaks are integrated, and the peak area is used with linear regression analysis from the calibration curves to determine the solubility values.^9^

#### ELog*D*_7.4_

The extrapolated Log*D*_7.4_ (ELog*D*_7.4_) analysis utilizes a Waters Acquity I Class Plus binary pump, autosampler, column compartment, and PDA detector with a binary solvent system. The aqueous mobile phase is composed of 1-octanol-saturated Milli-Q water with 20 mM MOPS, 0.15% *n*-decylamine, and pH adjusted to 7.4. The organic phase is composed of methanol with 0.25% 1-octanol. A flow rate of 0.4 mL/min with an Acquity UPLC CSH C18, 1.7 μm, 2.1 × 50 mm column at 37 °C was used for all runs. Three chromatograms are obtained for the calibration mixture, control mixture, and each compound with isocratic conditions at 55%, 60%, and 70% organic phase. The calibration mixture contains uracil and compounds with known Log*D*_7.4_ values. Uracil is used to determine the dead volume (dead time, *t*_0_), and the retention time (*t*_R_) for each compound is used to calculate the capacity factor (*k*) using the following equation:
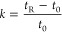
A plot with the Log*k* values from the calibration mixture vs the organic phase percentage allows for the extrapolation down to Log*k* at 0% organic phase, and then a calibration plot of Log*k*_0% organic phase_ vs the known Log*D*_7.4_ values allows for the linear extrapolation of ELog*D*_7.4_ values. The controls are used to confirm that the calibration curve was successful. Log*k* values are then determined for each test compound, extrapolated down to 0% organic phase, and then the Log*k*_0% organic phase_ vs known Log*D*_7.4_ values plot is used to determine the ELog*D*_7.4_ values. This chromatographic method is not suitable for the determination of ELog*D*_7.4_ values for acidic compounds.^10b,10c^

#### P-gp Efflux

##### Cell Culture

In-house MDCKII-MDR1 cells were cultured in media consisting of Dulbecco’s modified Eagle’s media (low glucose), 25 mM HEPES, 10% fetal bovine serum, 1% nonessential amino acids, 100 units/mL penicillin/streptomycin, and 4 mM G418 at 37 °C, 5% CO_2_, and 85% relative humidity. On day 1, MDCKII-MDR1 cells were seeded at a density of 45,000 cells/well onto Corning (Corning, NY) 24-well Transwell plates (0.4 μm pore size, 0.33 cm^2^ growth area) and placed in the cell culture incubators. The assay was performed on day 5. The Transwell plates received a fresh media change 1 day before the experiments to prevent cell starvation.

##### P-Glycoprotein Transwell Assay

All Transwell assays were performed in HBSS buffer. Transwell assays were performed at 5 μM concentrations of compounds. Transporter studies were initiated by adding dosing solutions into donor compartments and measuring the appearance of compounds in receiver compartments after 120 min. Before incubation, donor and receiver samples at 0 min were collected, and after 120 min of incubation, samples were collected for both donor and receiver chambers and crashed out with cold acetonitrile containing internal standard (50 nM carbamazepine). The plates were then centrifuged at 3000 RCF (4 °C) for 10 min, and the supernatant was transferred to a separate 96-well plate and diluted 1:1 with water and 0.2% formic acid for LC/MS/MS analysis. The cell monolayer integrity during the incubation with the test compounds was assessed after a 120 min assay duration by measuring lucifer yellow (100 μM) fluorescence in both donor and receiver chambers. Quinidine and propranolol were used as P-gp substrate and high-passive permeability control, respectively. All the data for controls were within the acceptable range. The % recovery for the test compounds was above 80%.

##### Data Analysis

To determine apparent permeability (*P*_app_), the following equation was used:
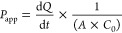
where d*Q*/d*t* is the rate of appearance of the test compounds in the receiver compartment, *A* is surface area of the membrane (0.33 cm^2^), and *C*_0_ is the initial concentration (0 min) of the test compounds in the donor compartment.

The ER was calculated by the following equation:
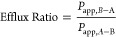
where *P*_app,B–A_ and *P*_app,A–B_ refers to the permeability in the direction of basolateral to apical (B-to-A) and apical to basolateral (A-to-B), respectively.

## Supporting Information Available

^1^H and ^13^C{^1^H} NMR spectra for all compounds, LCMS traces for **1**–**3**, and X-ray crystallography data (PDF)
NMR data files (ZIP)
