# Supplementary material for: Total Synthesis and Pharmacological Evaluation of Phochrodines A–C
Source: J Nat Prod. 2025 Apr 1;88(4):996–1003. doi: 10.1021/acs.jnatprod.5c00104 (PMC12038836; doi:10.1021/acs.jnatprod.5c00104)
Supplement: Supplementary file 1 — np5c00104_si_001.pdf [file np5c00104_si_001.pdf]

Supporting Information

Total Synthesis and Pharmacological Evaluation of Phochrodines A-C

Jacob L. Bouchard,[a,b] Sichen Chang,[a,b] Srinivasan Krishnan,[a,b] Christopher C. Presley,[a,b] Olivier Boutaud,[a,b] Nathan D. Schley,[c] Darren W. Engers,[a,b] Julie L. Engers,[a,b] Craig W. Lindsley,[a,b,c,d] Aaron M. Bender [a,b,*]

a. Warren Center for Neuroscience Drug Discovery, Vanderbilt University, Nashville, Tennessee 37232, United States
b. Department of Pharmacology, Vanderbilt University, Nashville, Tennessee 37232, United States
c. Department of Chemistry, Vanderbilt University, Nashville, Tennessee 37240, United States
d. Department of Biochemistry, Vanderbilt University, Nashville, Tennessee 37205, United States

*Corresponding author email: aaron.bender@vanderbilt.edu

Copies of NMR Spectra S2

LCMS Traces for Phochrodines A-C (**1-3**) S11

Crystallography Data for Phochrodine A (**1**) S14

Copies of NMR Spectra

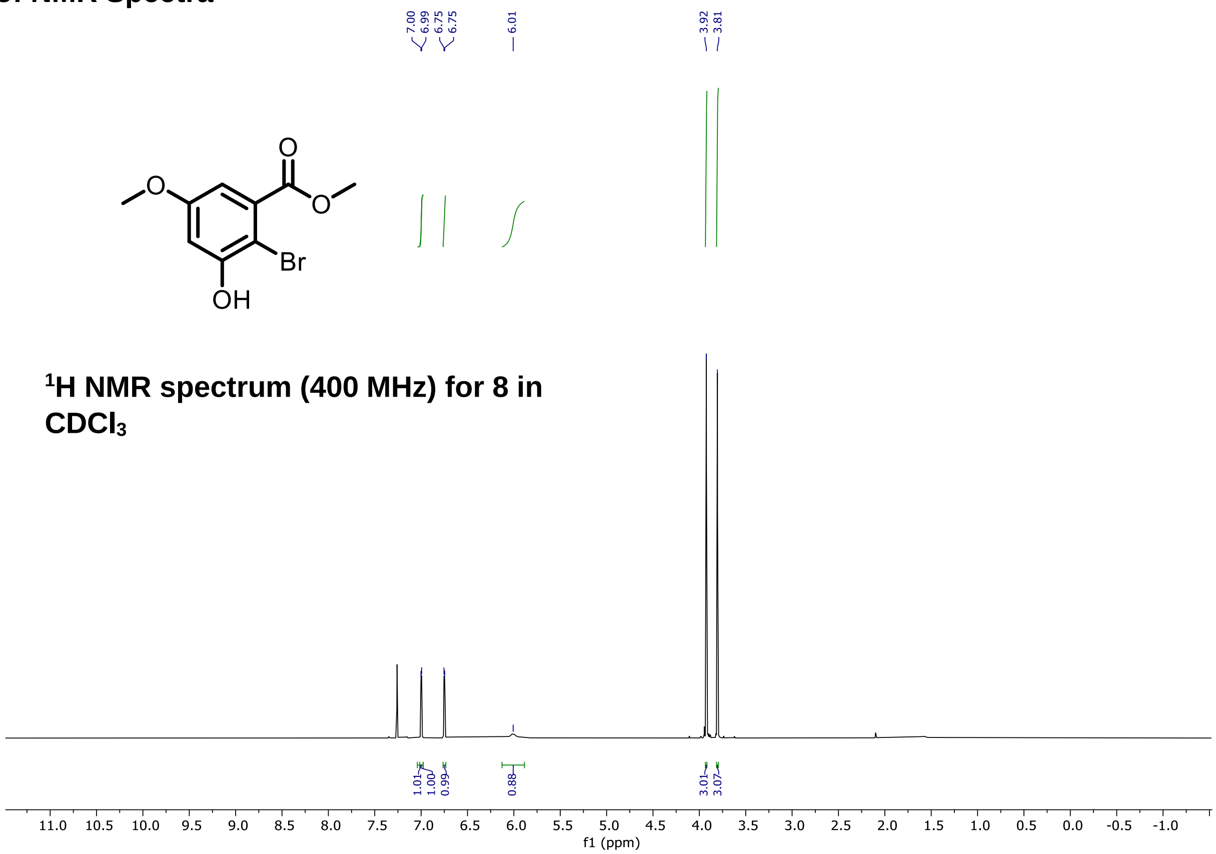

^{1}H NMR spectrum (400 MHz) for 8 in $CDCl_3$

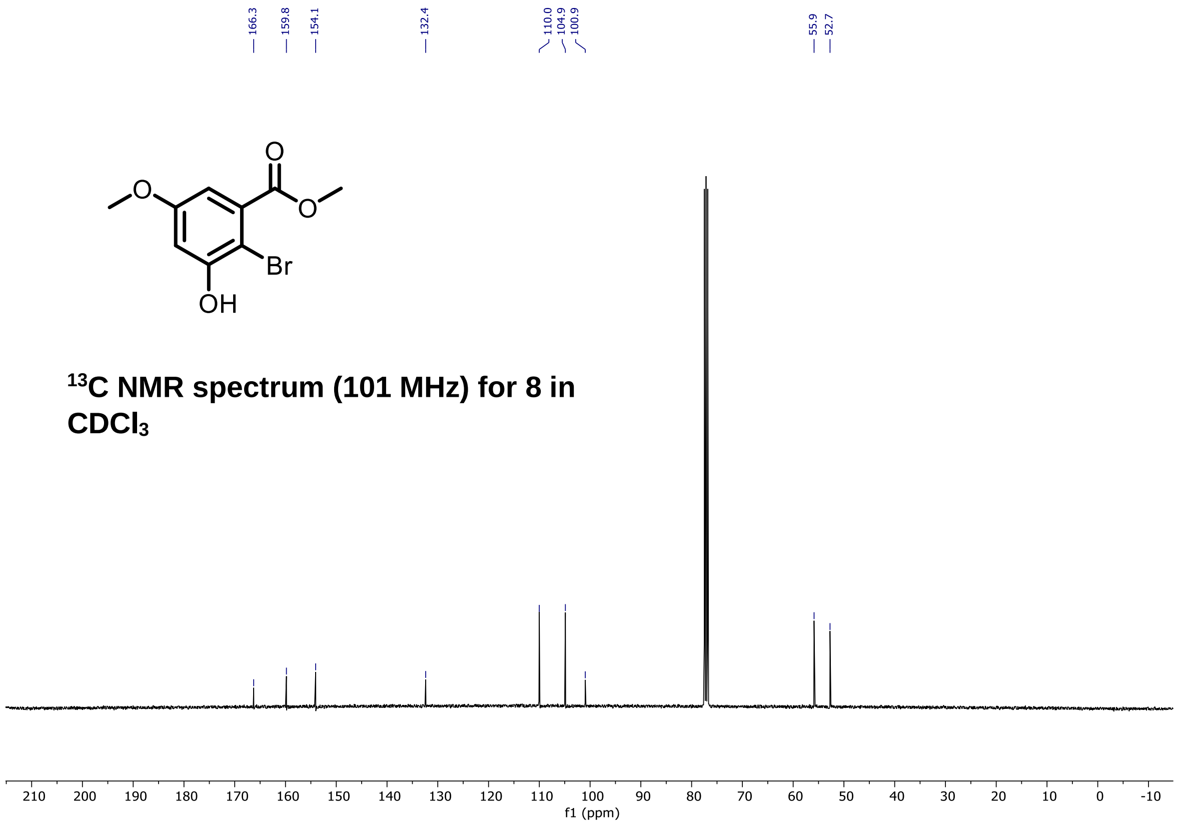

^{13}C NMR spectrum (101 MHz) for 8 in $CDCl_3$

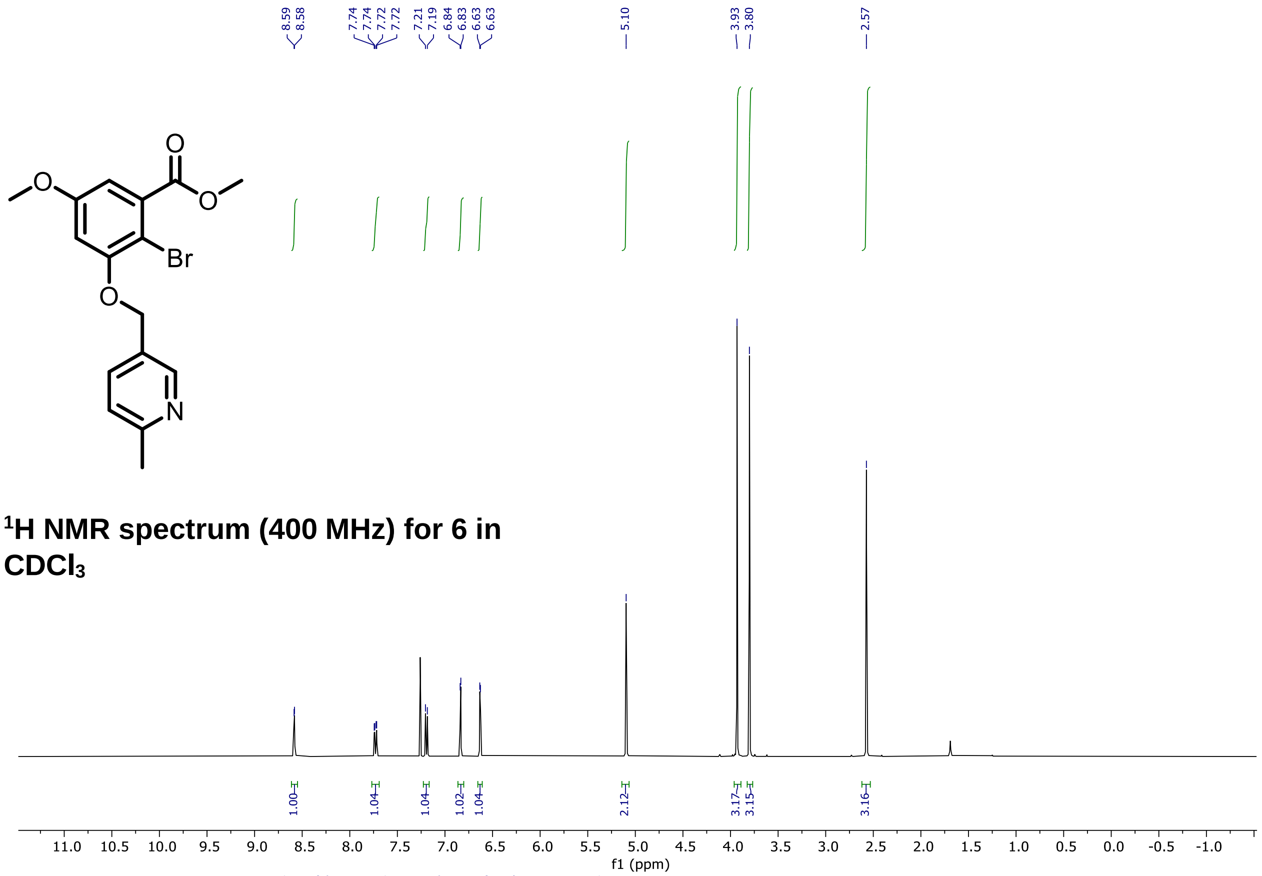

^{1}H NMR spectrum (400 MHz) for 6 in $CDCl_3$

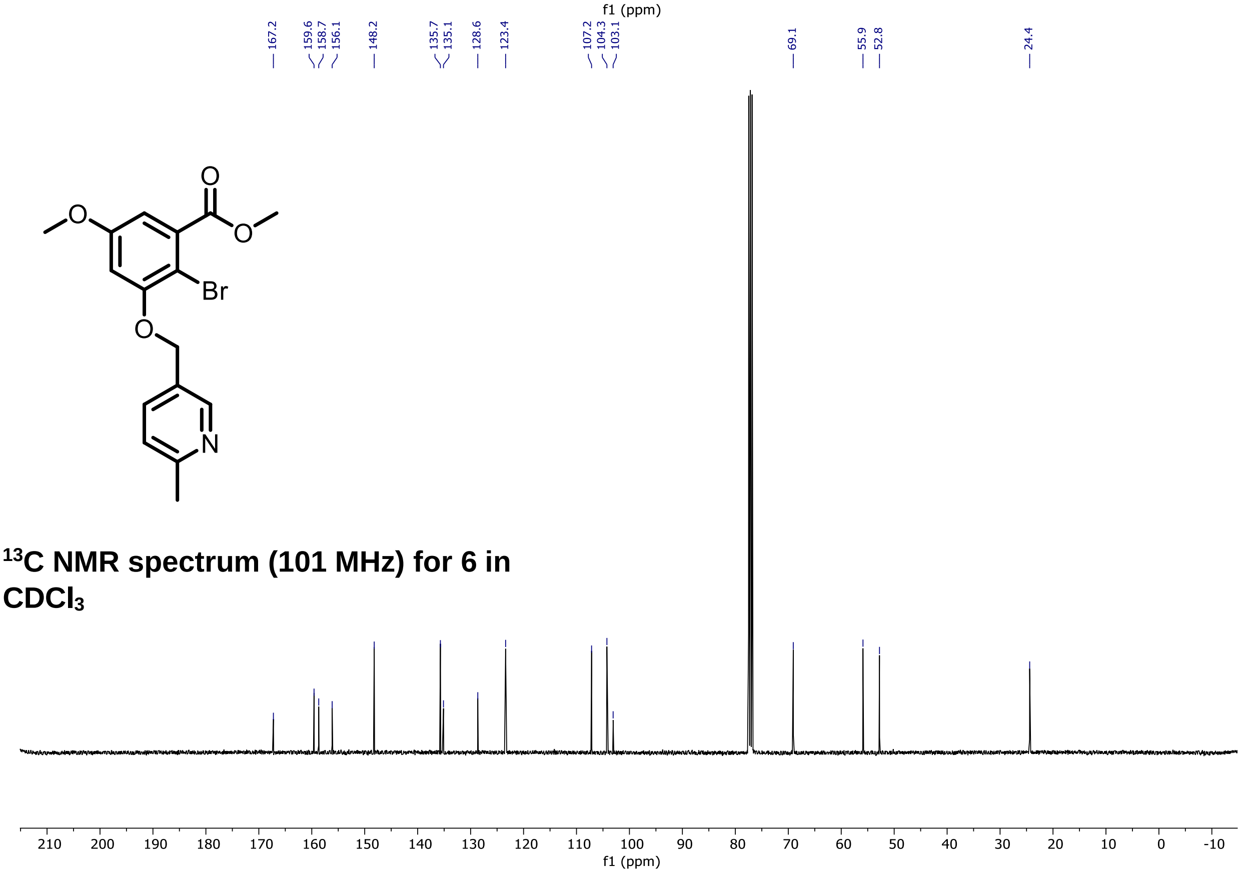

^{13}C NMR spectrum (101 MHz) for 6 in $CDCl_3$

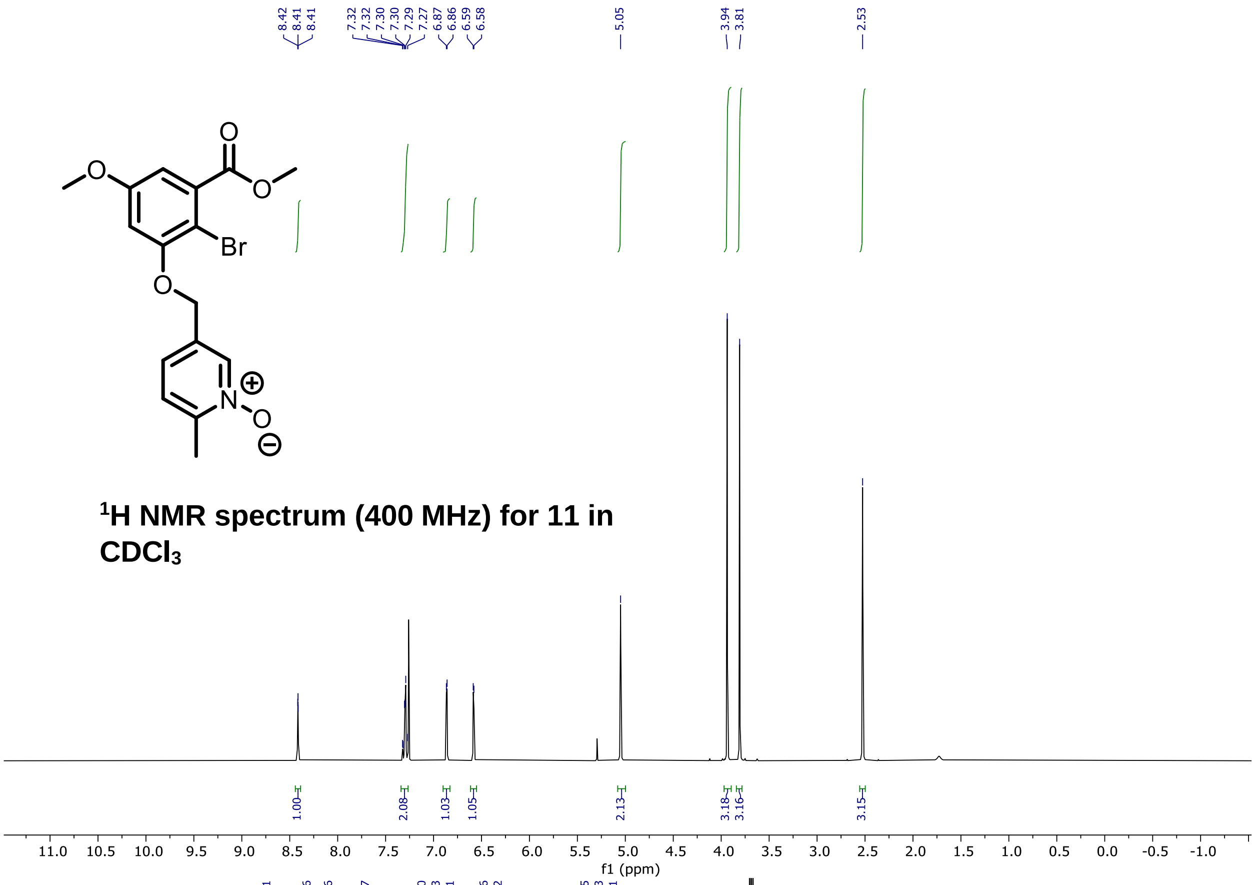
8.42
8.41
8.41
7.32
7.32
7.30
7.30
7.29
7.27
6.87
6.86
6.59
6.58
5.05
3.94
3.81
2.53
Br
^{1}H NMR spectrum (400 MHz) for 11 in $CDCl_3$
1.00
2.08
1.03
1.05
2.13
3.18
3.16
3.15
f1 (ppm)

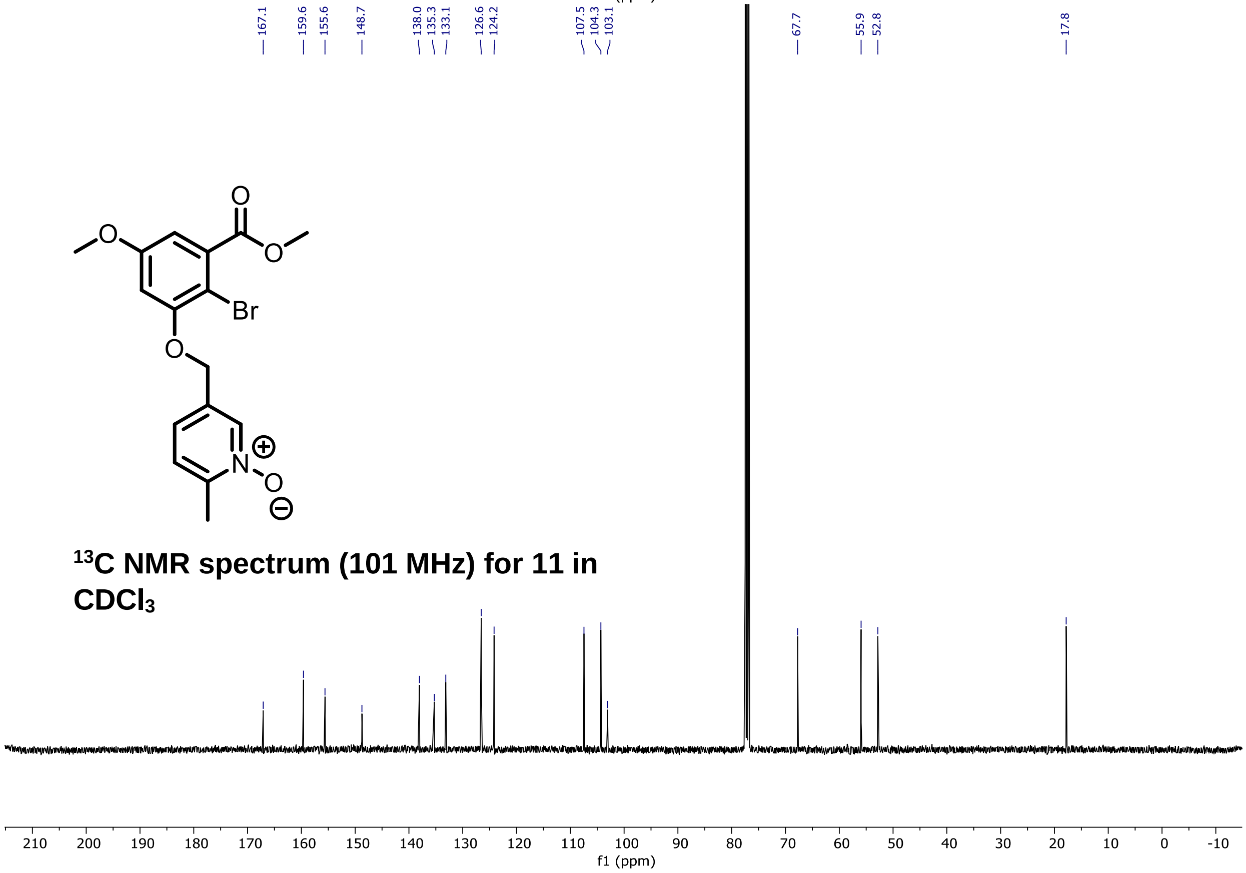
167.1
159.6
155.6
148.7
138.0
135.3
133.1
126.6
124.2
107.5
104.3
103.1
67.7
55.9
52.8
17.8
Br
^{13}C NMR spectrum (101 MHz) for 11 in $CDCl_3$
f1 (ppm)

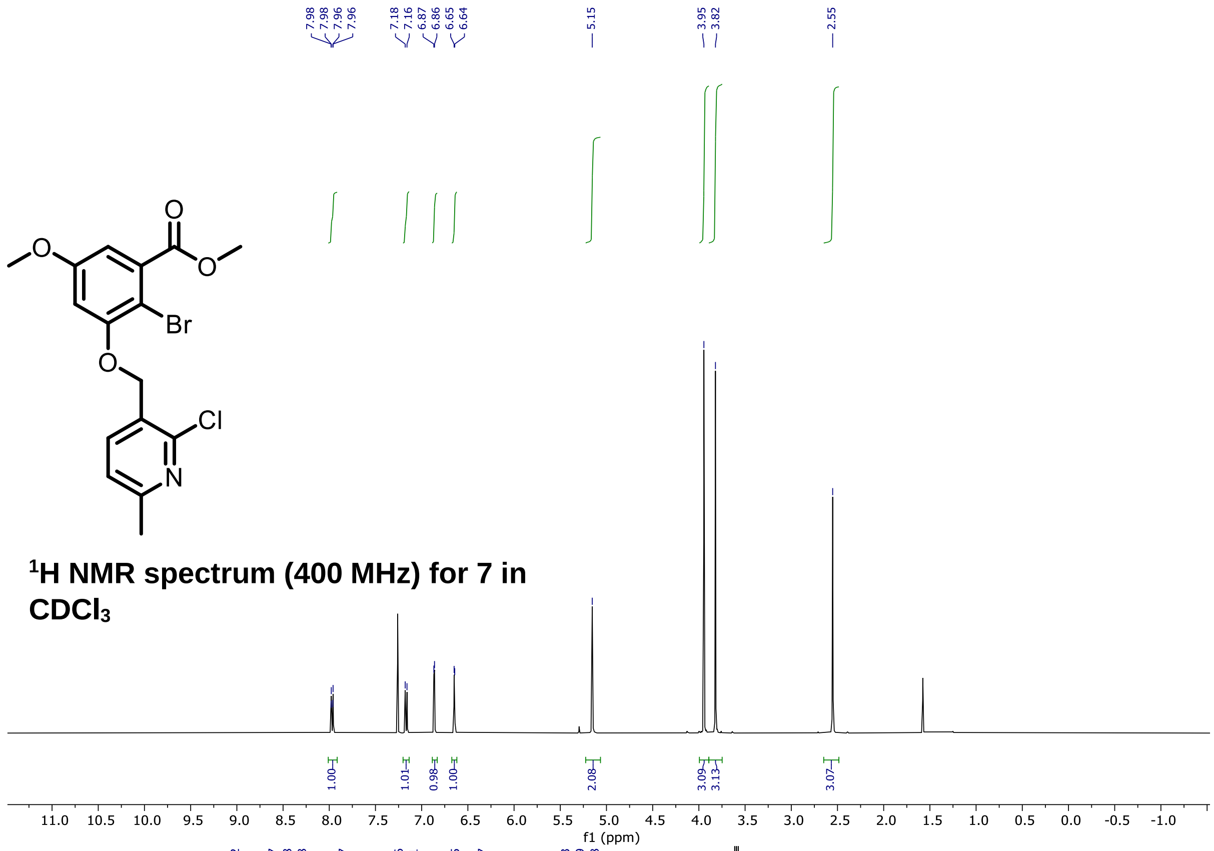

^{1}H NMR spectrum (400 MHz) for 7 in $CDCl_3$

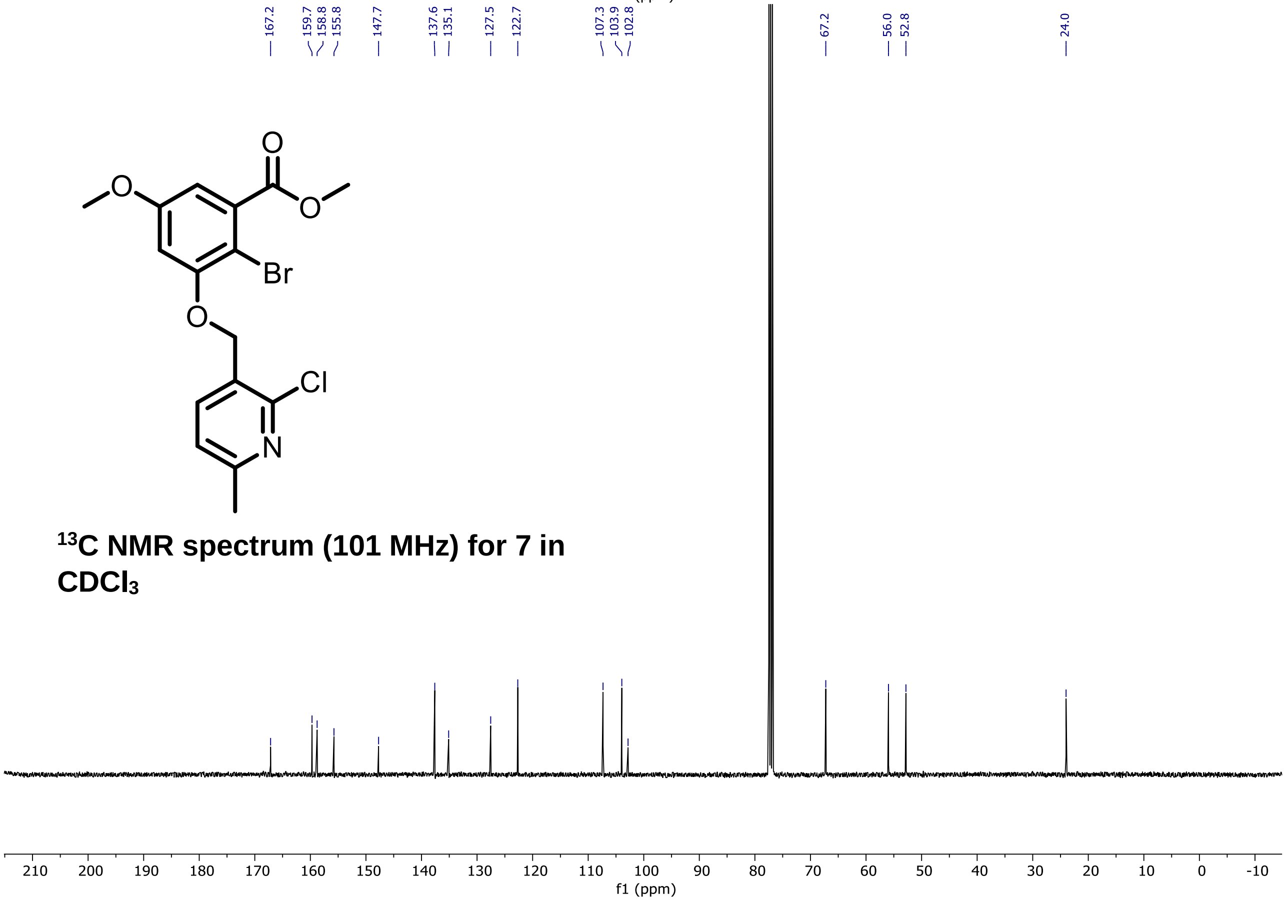

^{13}C NMR spectrum (101 MHz) for 7 in $CDCl_3$

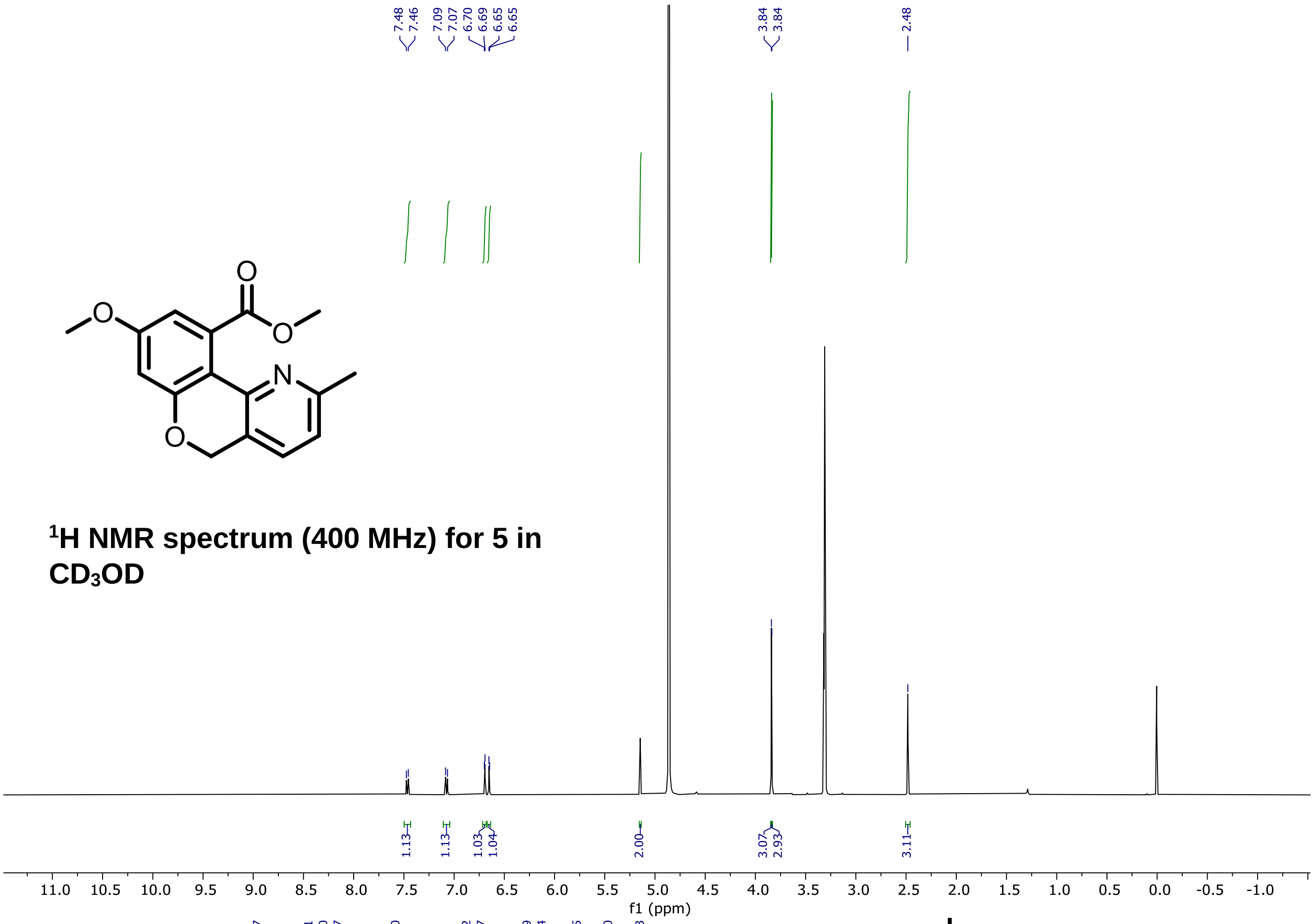

^{1}H NMR spectrum (400 MHz) for 5 in CD_3OD

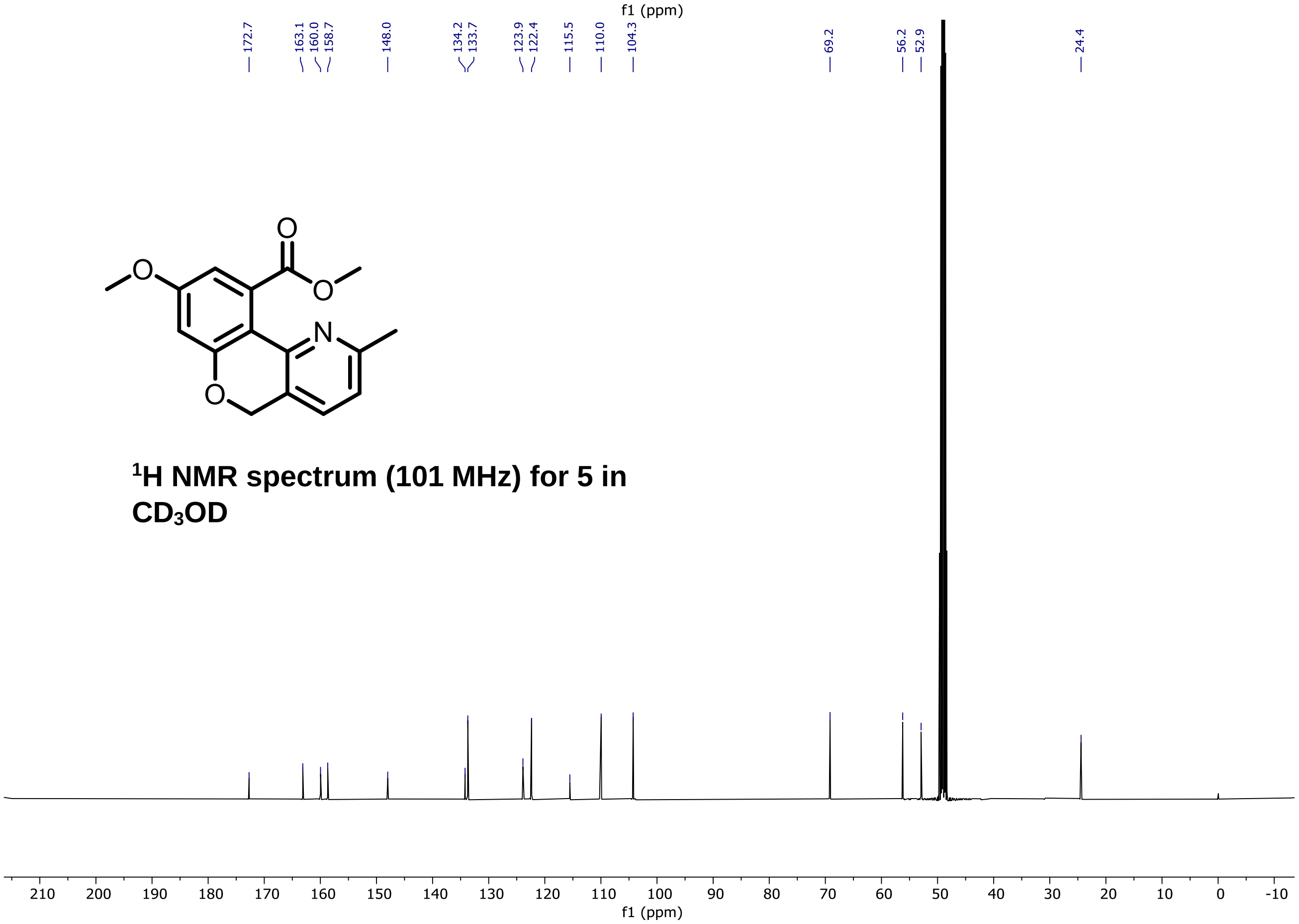

^{1}H NMR spectrum (101 MHz) for 5 in CD_3OD

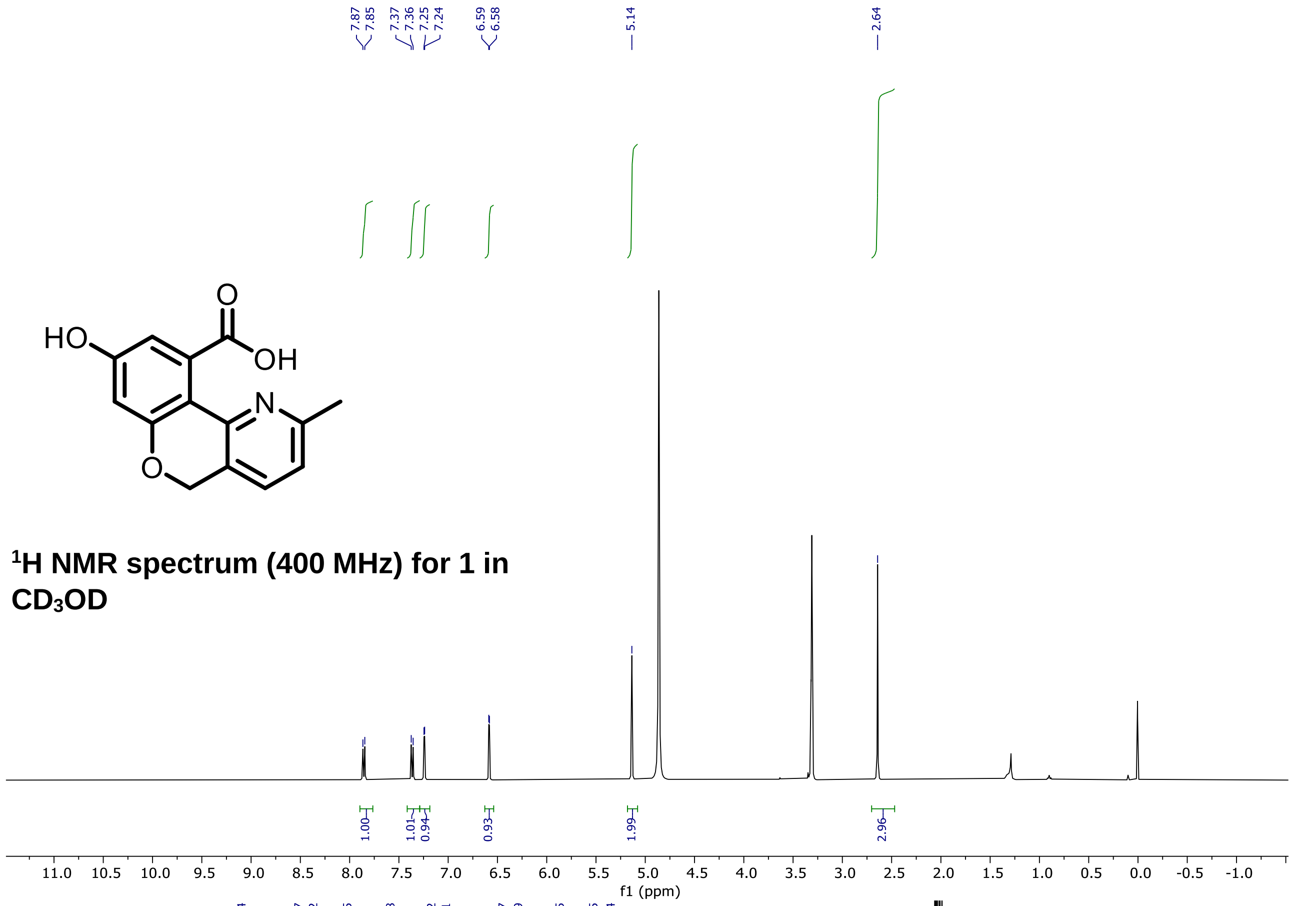

^{1}H NMR spectrum (400 MHz) for 1 in CD_3OD

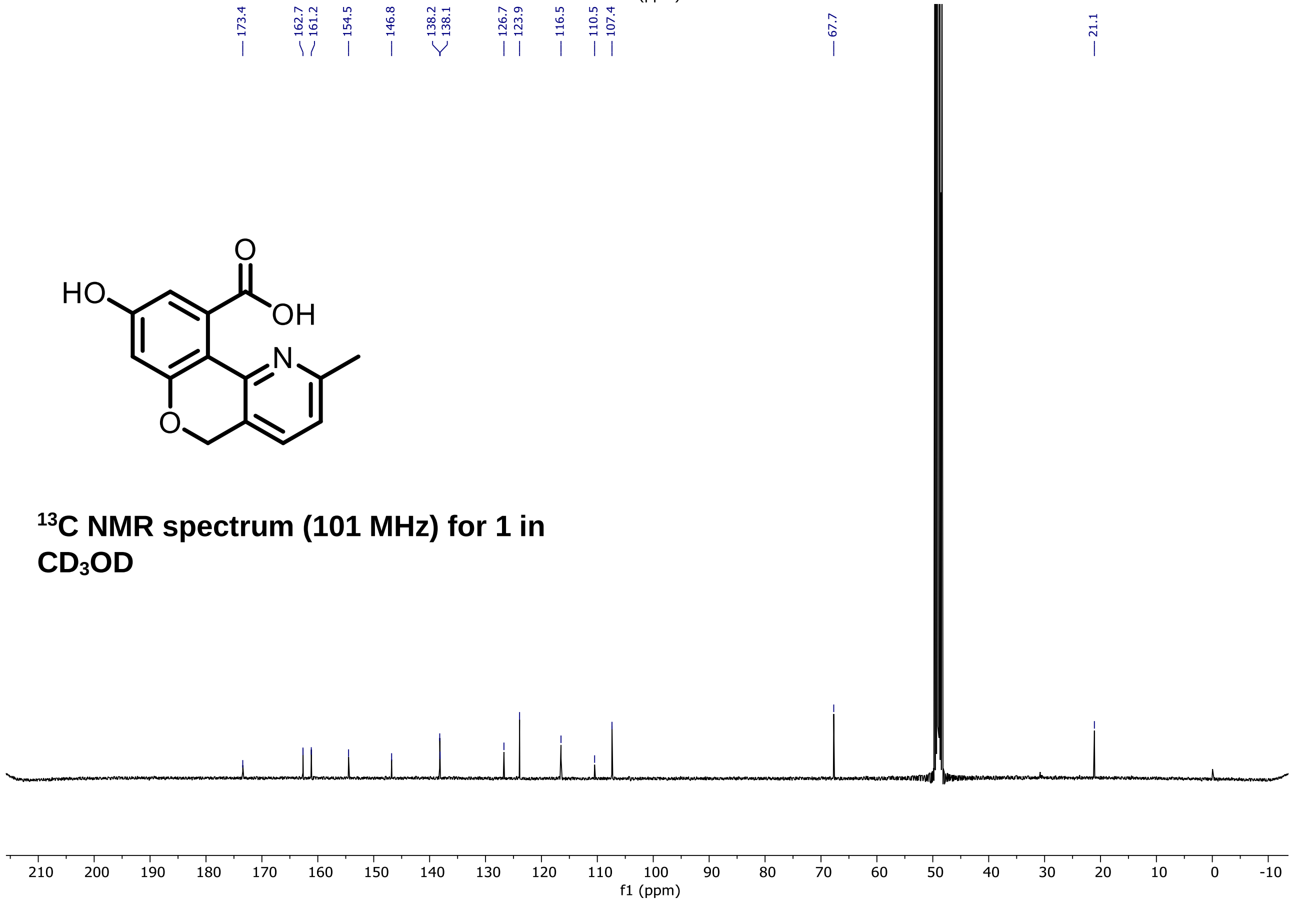

^{13}C NMR spectrum (101 MHz) for 1 in CD_3OD

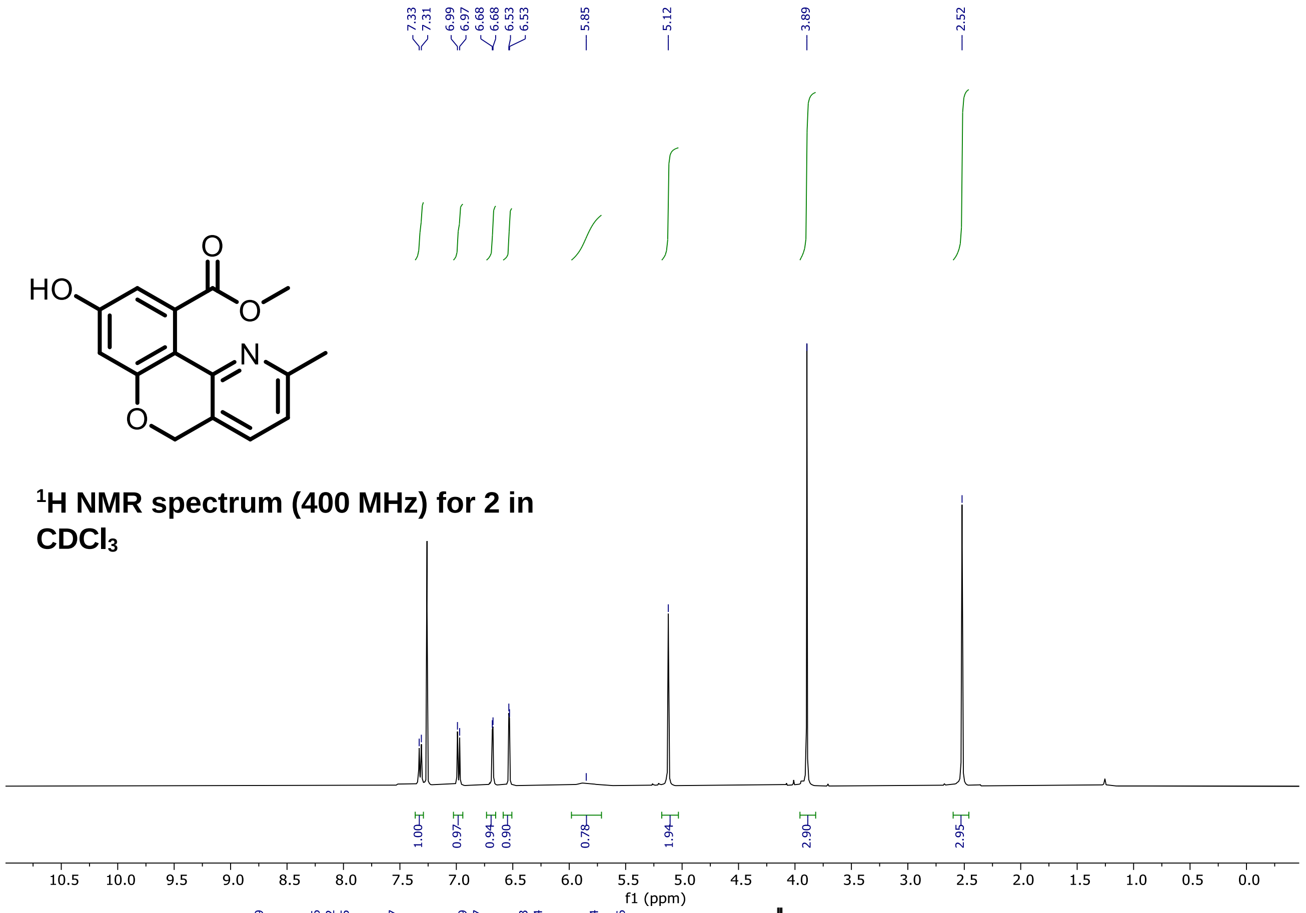

^{1}H NMR spectrum (400 MHz) for 2 in $CDCl_3$

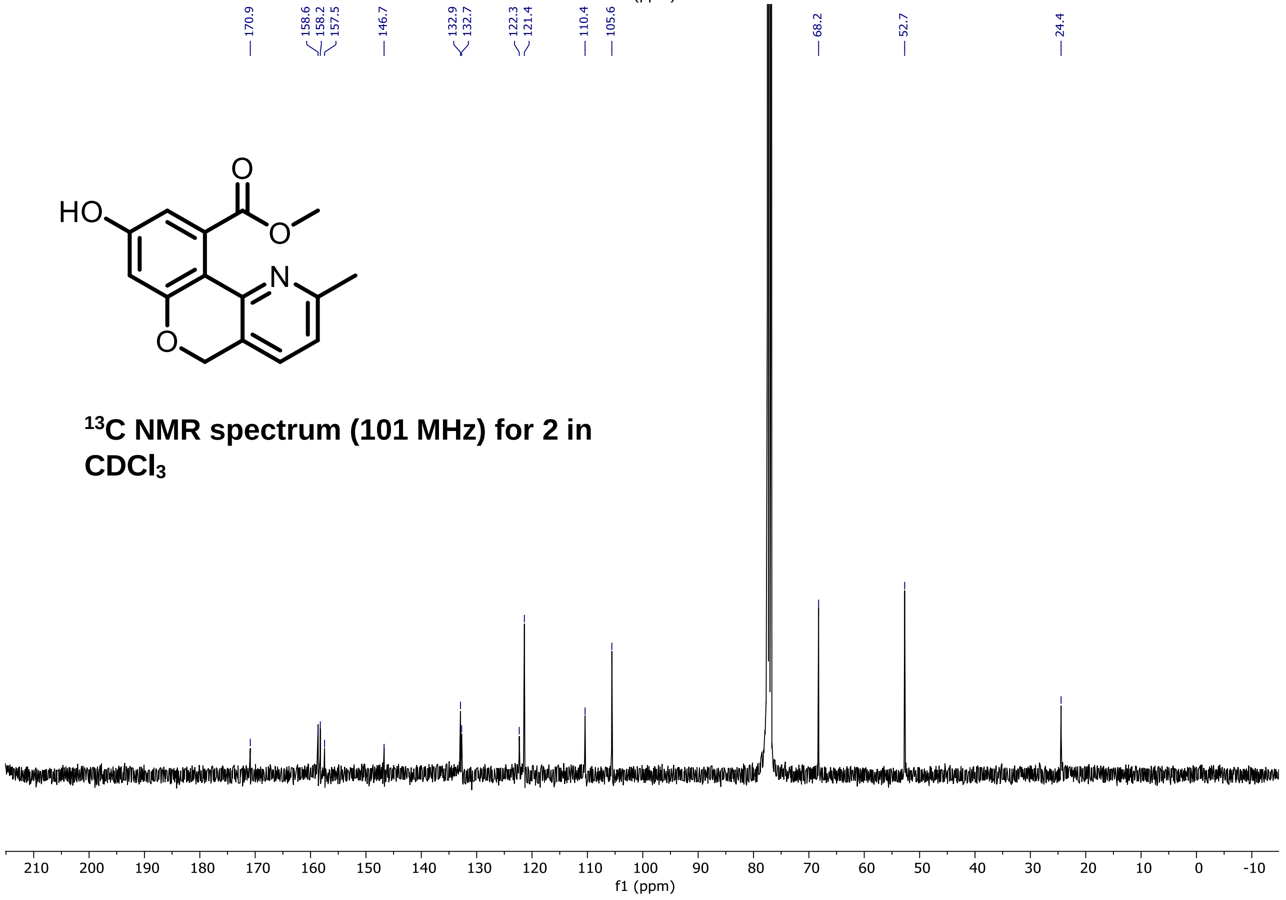

^{13}C NMR spectrum (101 MHz) for 2 in $CDCl_3$

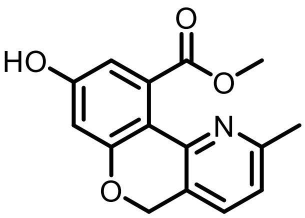

^{13}C NMR spectrum (101 MHz) for 2 in CD_3OD

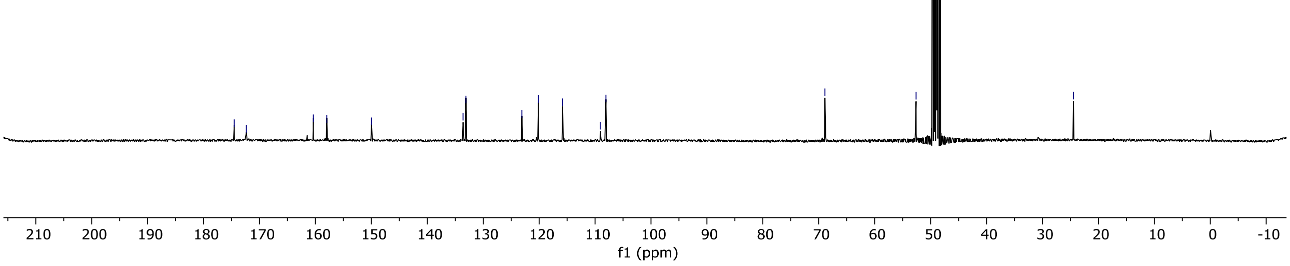

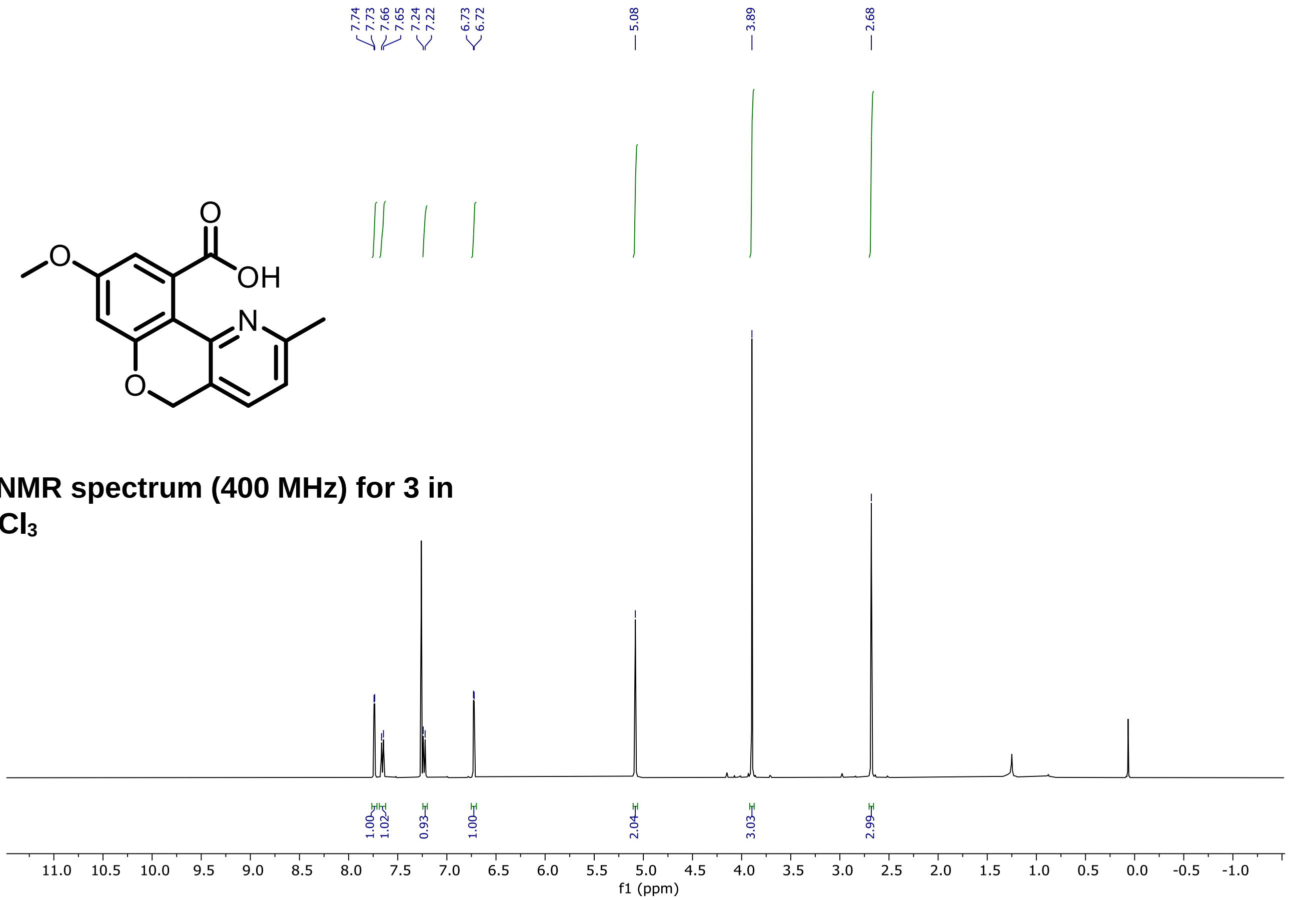

^{1}H NMR spectrum (400 MHz) for 3 in $CDCl_3$

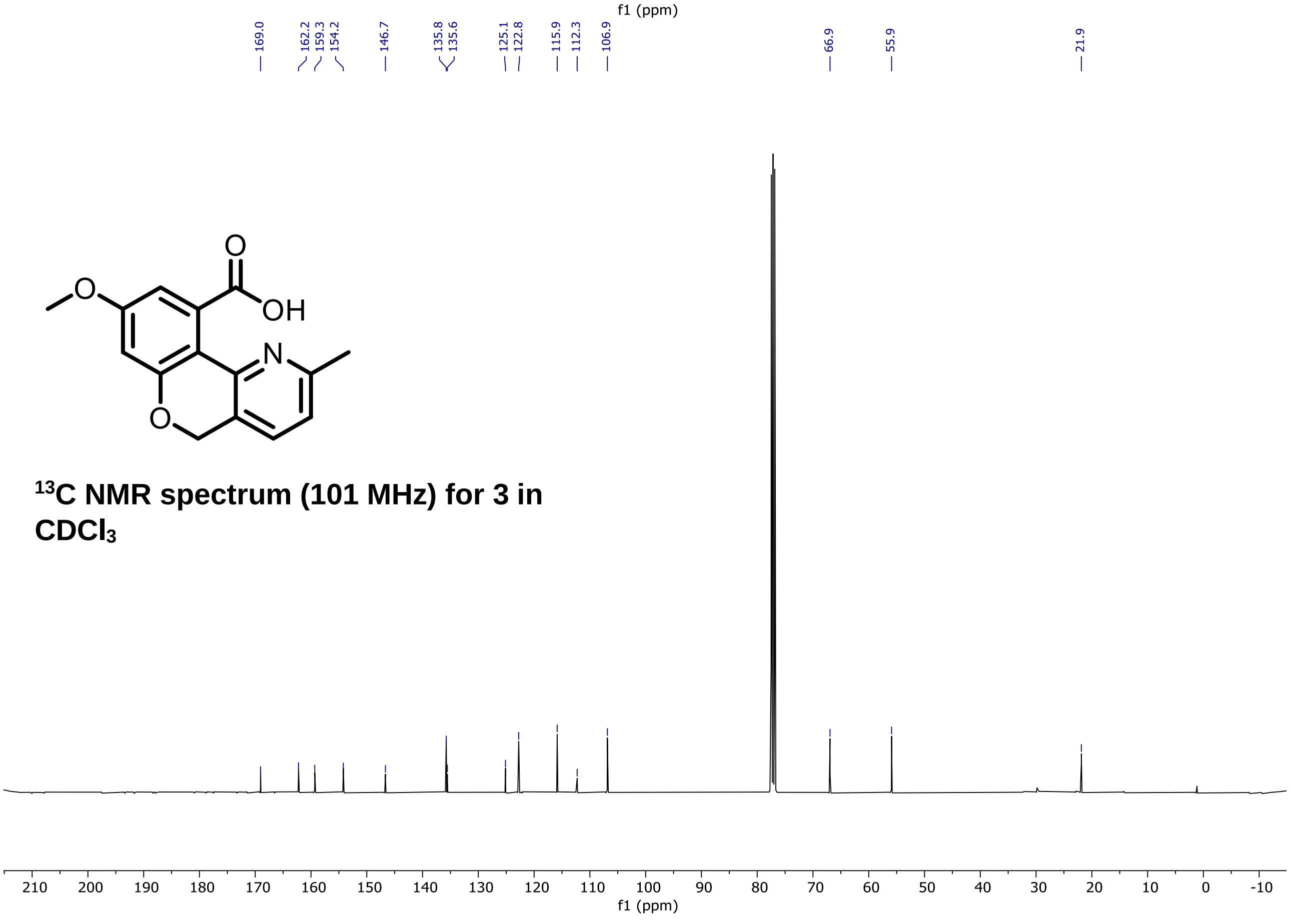

^{13}C NMR spectrum (101 MHz) for 3 in $CDCl_3$

LCMS Traces for Phochrodines A-C (1-3)

Phochrodine A (**1**)

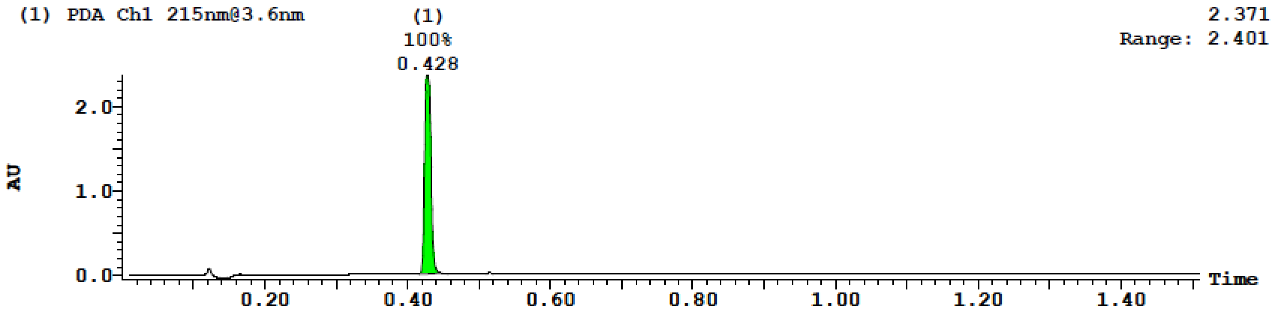

Peak Number	Time	Area %Total	BPM	Mass Found
1	0.428	100.00	258	257.0000

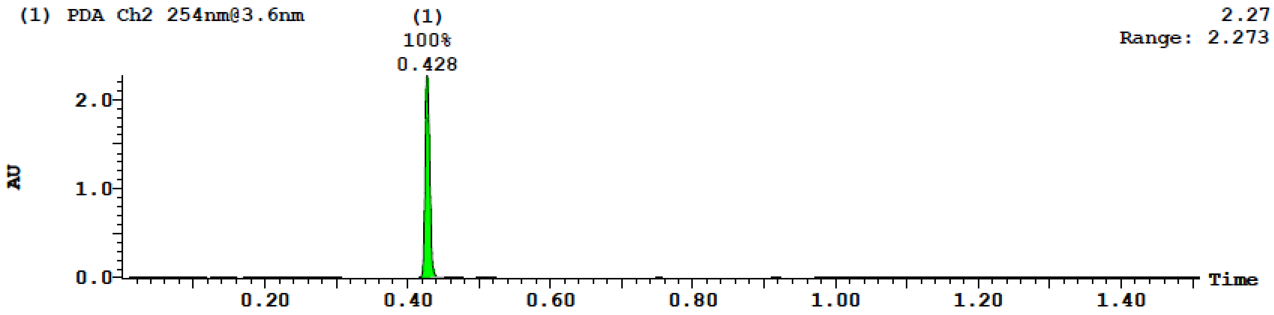

Peak Number	Time	Area %Total	BPM	Mass Found
1	0.428	100.00	258	257.0000

Peak ID	Time
1	0.428

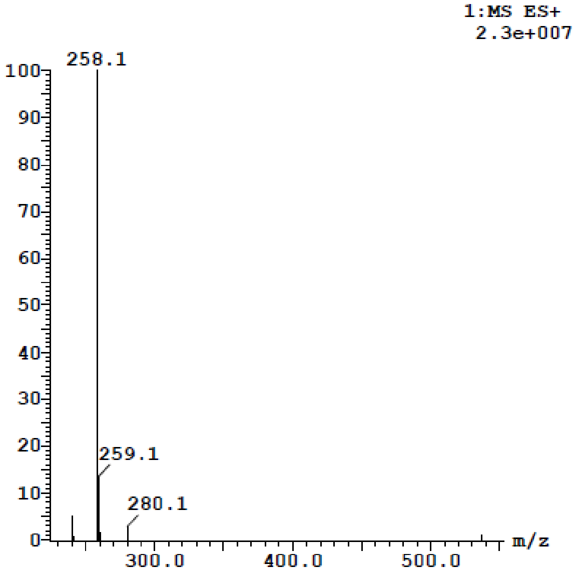

Phochrodine B (**2**)

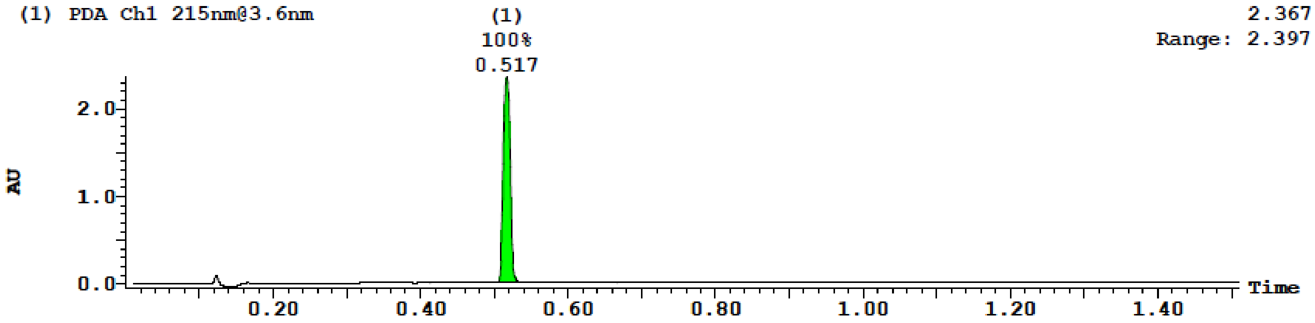

Peak Number	Time	Area %Total	BPM	Mass Found
1	0.517	100.00	272	271.0000

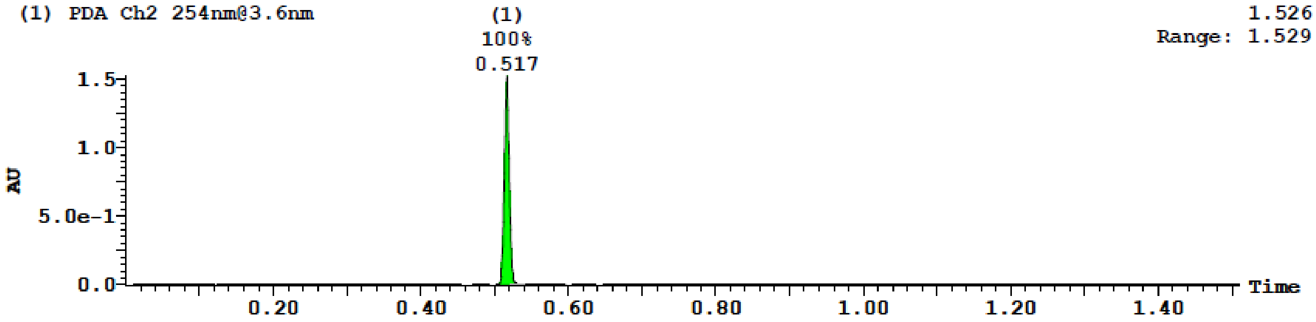

Peak Number	Time	Area %Total	BPM	Mass Found
1	0.517	100.00	272	271.0000

Peak ID	Time
1	0.517

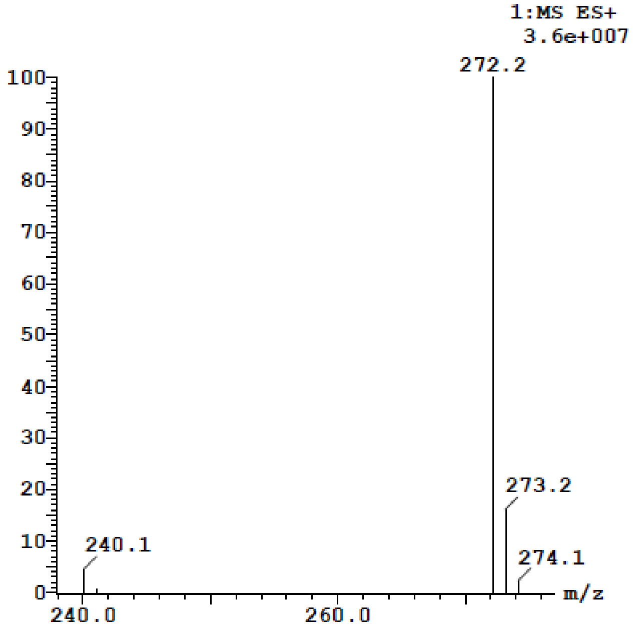

Phochrodine C (**3**)

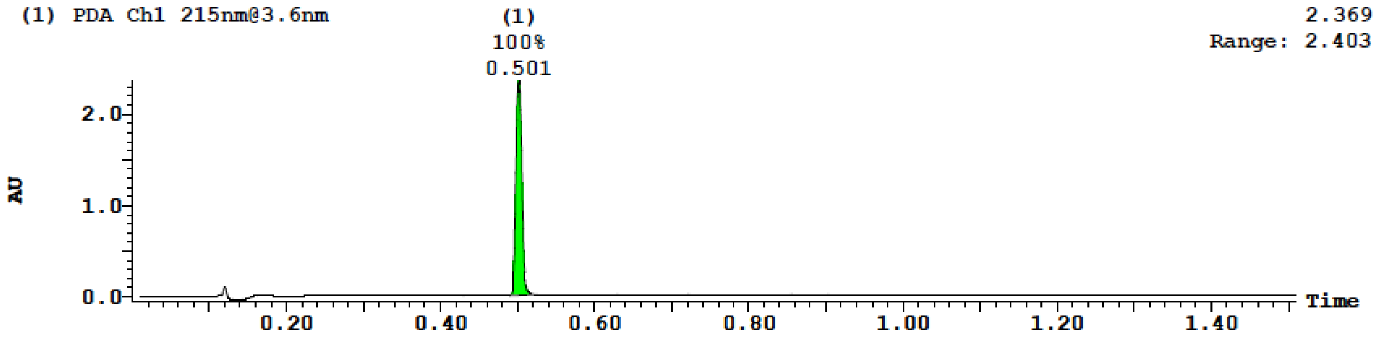

Peak Number	Time	Area %Total	BPM	Mass Found
1	0.501	100.00	272	271.0000

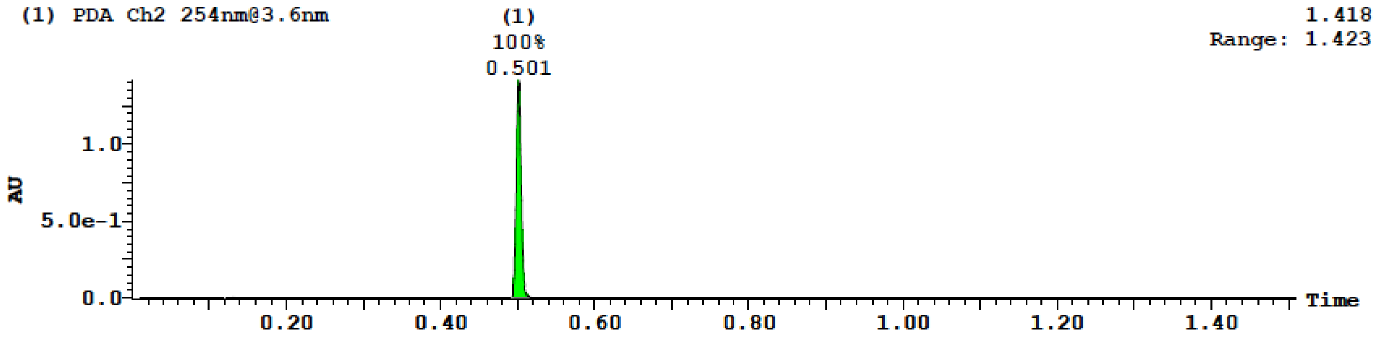

Peak Number	Time	Area %Total	BPM	Mass Found
1	0.501	100.00	272	271.0000

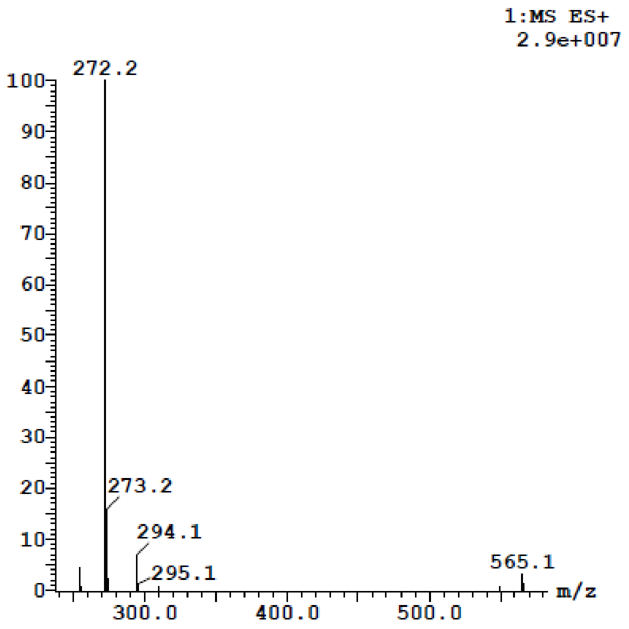

Crystallography Data for Phochrodine A (1)

Crystals of **1** were grown via evaporation of a MeOH/EtOAc mixture at room temperature.

Details of crystallographic refinement

General Methods. A suitable crystal of each sample was selected for analysis and mounted in a polyimide loop. Crystal samples were handled under immersion oil and quickly transferred to a cold nitrogen stream. All measurements were made on a Rigaku Oxford Diffraction Supernova Eos CCD with filtered Cu-Kα radiation at a temperature of 100 K. Using Olex2,[1] the structure was solved with the ShelXT structure solution program using Direct Methods and refined with the ShelXL refinement package[2] using Least Squares minimization.

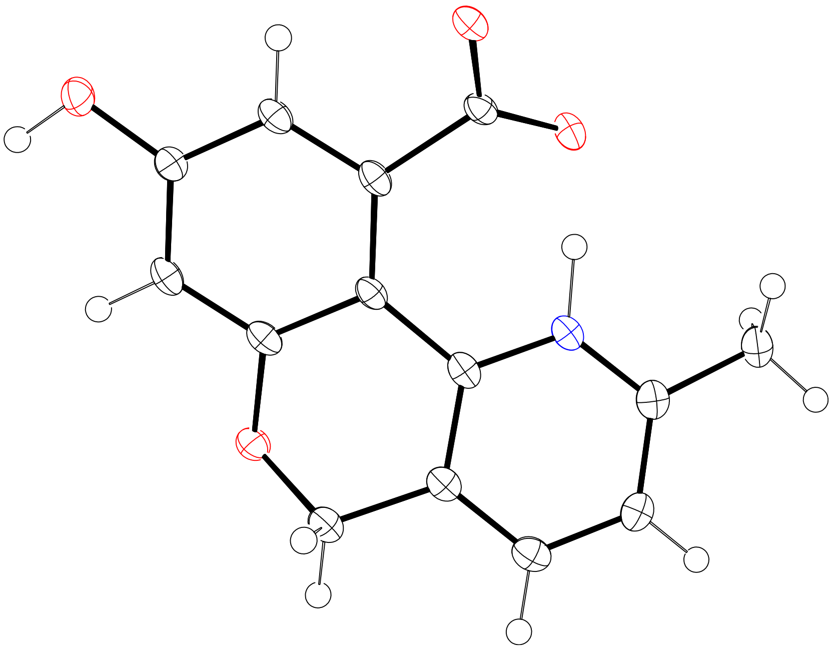

The hydrogen atoms on oxygen or nitrogen were located in the difference map and refined without restraint, with the exception of the water molecule, which was refined as a rigid body.

(1) Dolomanov, O. V.; Bourhis, L. J.; Gildea, R. J.; Howard, J. A. K.; Puschmann, H., OLEX2: A Complete Structure Solution, Refinement and Analysis Program. *J. Appl. Crystallogr*. **2009**, *42*, 339-341.

(2) Sheldrick, G., A Short History of SHELX. *Acta Crystallogr. Sect. A* **2008**, *64*, 112-122.

Table S1. Crystal data and structure refinement for 1

Identification code	jlb-3-81_2	
Empirical formula	C14 H12 N O4.50	
Formula weight	266.25	
Temperature	100.00(10) K	
Wavelength	1.54184 Å	
Crystal system	Monoclinic	
Space group	P 1 21/c 1	
Unit cell dimensions	a = 17.5222(8) Å	α = 90°
	b = 7.1156(3) Å	β = 94.967(4)°
	c = 18.8044(8) Å	γ = 90°
Volume	2335.75(18) Å^3	
Z	8	
Density (calculated)	1.514 Mg/m^3	
Absorption coefficient	0.962 mm^{-1}	
F(000)	1112	
Crystal size	0.39 x 0.17 x 0.02 mm^3	
Theta range for data collection	2.531 to 71.522°.	
Index ranges	-21<=h<=19, -8<=k<=5, -22<=l<=22	
Reflections collected	13290	
Independent reflections	4461 [R(int) = 0.0386]	
Completeness to theta = 67.684°	99.9 %	
Absorption correction	Gaussian	
Max. and min. transmission	1.000 and 0.628	
Refinement method	Full-matrix least-squares on F^2	
Data / restraints / parameters	4461 / 0 / 373	
Goodness-of-fit on F^2	1.048	
Final R indices [I>2sigma(I)]	R1 = 0.0560, wR2 = 0.1446	
R indices (all data)	R1 = 0.0716, wR2 = 0.1586	
Extinction coefficient	n/a	
Largest diff. peak and hole	1.404 and -0.308 e/Å^{-3}	
